# *In silico* Method in CRISPR/Cas System: An Expedite and Powerful Booster

**DOI:** 10.3389/fonc.2020.584404

**Published:** 2020-10-02

**Authors:** Yuwei Zhang, Guofang Zhao, Fatma Yislam Hadi Ahmed, Tianfei Yi, Shiyun Hu, Ting Cai, Qi Liao

**Affiliations:** ^1^Hwa Mei Hospital, University of Chinese Academy of Science, Ningbo, China; ^2^Zhejiang Key Laboratory of Pathophysiology, Department of Preventative Medicine, Medical School of Ningbo University, Ningbo, China; ^3^Ningbo Institute of Life and Health Industry, University of Chinese Academy of Sciences, Ningbo, China

**Keywords:** CRISPR/Cas system, *In silico* methods, CRISPR/Cas system identification, guide RNA design, post-experimental assistance

## Abstract

The CRISPR/Cas system has stood in the center of attention in the last few years as a revolutionary gene editing tool with a wide application to investigate gene functions. However, the labor-intensive workflow requires a sophisticated pre-experimental and post-experimental analysis, thus becoming one of the hindrances for the further popularization of practical applications. Recently, the increasing emergence and advancement of the *in silico* methods play a formidable role to support and boost experimental work. However, various tools based on distinctive design principles and frameworks harbor unique characteristics that are likely to confuse users about how to choose the most appropriate one for their purpose. In this review, we will present a comprehensive overview and comparisons on the *in silico* methods from the aspects of CRISPR/Cas system identification, guide RNA design, and post-experimental assistance. Furthermore, we establish the hypotheses in light of the new trends around the technical optimization and hope to provide significant clues for future tools development.

## Introduction

**Figure 1:**
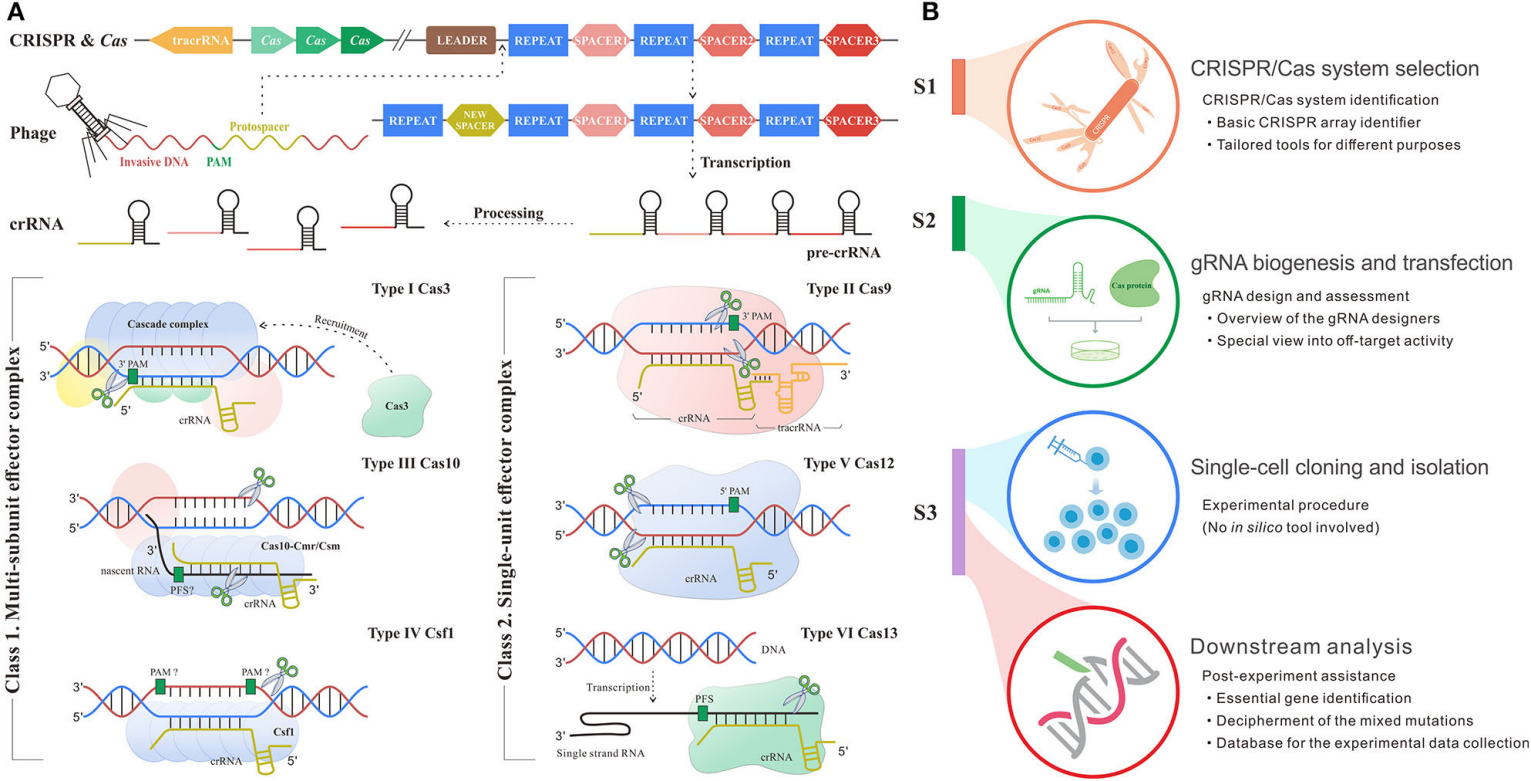
Schematic diagram shows the mechanism and workflow of CRISPR/Cas adaptive immune system. **(A)** The mechanism of CRISPR/Cas system. S1: Adaptation stage. The invasive DNA sequence produced by phage is cleaved and incorporated into the start of a CRISPR array comprised of a string of spacers flanked by repeats, forming a new spacer downstream leader. S2: CRISPR RNA (crRNA) biogenesis stage. The precursor of crRNA transcribed from CRISPR array is further processed into mature crRNA, which carries the genetic information from spacer. S3: Interference stage across six main types of systems. In type I system (signature protein: Cas3), the multimeric effector, Cascade, binds to target DNA complementary to crRNA and then recruits Cas3 to generate the single-strand nick. Type II system (signature protein: Cas9) encodes tracRNA to hybridize with crRNA and form a dual tracRNA:crRNA complex, which guides Cas9 enzyme to the target and thus generates blunt double-strand breaks (DSBs). In type III system (signature protein: Cas10), Cas10-Cmr/Csm complex recognizes the nascent target RNA following by the new enzymatic activity for complementary DNA cleavage. Type IV system (signature protein: Csf1) remains mostly unknown, although current research had demonstrated the crRNA maturation and proved its evolutionary connection with type I system (15). Type V system (signature protein: Cas12) solely relies on the formation of a binary complex between crRNA and Cas12 enzyme to identify target sequence and triggers staggered DSBs. In type VI system (signature protein: Cas13), crRNA binds to single-strand RNA through the protospacer flanking sequence (PFS) reorganization and guides Cas13 to realize the cleavage. **(B)** The workflow of CRISPR/Cas-mediated gene editing includes CRISPR/Cas system selection, guide RNA (gRNA) biogenesis and transfection, single-cell cloning and isolation, and downstream analysis. The subheadings under the main title represent the processes where *in silico* methods are involved. The flow linking the left and right panels represents the correspondence. For example, red flow shows that the implement of downstream analysis corresponds to the stage after CRISPR inference.

The mysterious veil of the genome and transcriptome in diverse organisms is being uncovered owing to contributive sequencing efforts. Even so, the functions of most genes remain unknown (1). The toughest challenge has been to associate phenotype changes to alterations on genetic layers. The state-of-the-art CRISPR/Cas system for genetic manipulation is an emerging tool to solve this nerve-wracking problem (2). CRISPR/Cas system is developed from a prokaryotic adaptive immune defense mechanism against the exogenous nucleic acids in archaea and bacteria (3), which follows a base-pairing rule between target and guide RNA (gRNA). The role of gRNA is to steer Cas enzyme to the custom positions in the presence of a protospacer adjacent motif (PAM) or protospacer flanking sequence (PFS) (4). PAM/PFS is a recognizable component following the target sites that enables precise cleavages on exogenous nucleic acids complementary to gRNA. In different types of CRISPR/Cas systems, gRNA could be the CRISPR RNA (crRNA), a kind of short non-coding RNAs derived from CRISPR arrays, or the synthetic formed by crRNA and trans-activating crRNA (tracrRNA). Besides, the category of CRISPR/Cas systems can be divided into two classes and subdivided into six types and 30 subtypes by different kinds of Cas effector module organizations, the position of the CRISPR array and acquisition module (5). As shown in Figure 1A, type I, III, and IV CRISPR/Cas systems have multi-subunit effector complexes and thereby collectively belong to class 1, while class 2 containing type II, V, and VI systems has a simpler architecture composed of only one protein effector (6–8).

Up to now, CRISPR/Cas system has been extensively applied in fundamental studies (9) as well as clinical practices across multiple diseases (10, 11). Of note, the discovery and implementation of CRISPR/Cas system require an intricate workflow (Figure 1B) including CRISPR/Cas system identification and selection, gRNA design, transfection, single-cell clone establishment, clone screening, and systematic mutation analysis (12–14). Each step expends considerable time, money, and manpower. Fortunately, the advance in computer science creates scope for remedying the deficiency and fueling the overall procedure (Figure 1B). *In silico* methods based on different algorithms and frameworks harbor different merits and are appropriate for diverse applications. Even though a variety of *in silico* tools goes on a growth spurt over recent years, there is a lack of a comprehensive summary for their roles in the overall procedure from system identification to application, so that many biological researchers are likely lost in the selection of suitable tools for their given intention. Therefore, it is necessary and urgent to make an explicit review of the existing tools.

In this review, we aim to summarize the released *in silico* methods from three major aspects (CRISPR/Cas system identification, gRNA design, and post-experimental assistance), discuss the relative merits, expound their applicability for various purposes, and put forward the possible assumptions for further improvements. We believe that our review is capable of elaborating on the roles of *in silico* toolkits in CRISPR/Cas system to formulate meaningful guidance for biological researchers and even provide significant clues for future tools development.

## CRISPR/Cas System Identification

At the phase of adaptation, bacteria copy a DNA segment (protospacer) from the invasive phages or plasmids and paste it to the start of the CRISPR array downstream of the leader sequence as a new spacer (Figure 1A) (3, 16, 17). CRISPR arrays are then transcribed and processed into crRNAs that possess partial genetic information of the invasive DNA and thus are able to form gRNA or directly guide Cas protein to the planned position (6). Since crRNAs and Cas protein, respectively, take full control of the specificity and editing efficiency of CRISPR/Cas systems, identification and classification of CRISPR/Cas system composed of different types of crRNAs and Cas proteins must be the most fundamental prerequisite for the downstream application.

### Recognition of CRISPR Arrays That Generate crRNAs

The most important component of CRISPR/Cas system, crRNA, is generated from CRISPR arrays (Figure 1A). Therefore, recognition of efficient CRISPR arrays largely determines the engineering specificity in the application. Until now, a variety of computational methods have been proposed to recognize CRISPR arrays using sequence information. One of the earliest tools, PatScan (18), was developed long before CRISPR/Cas system was applied in gene editing, which searches for the fragments homologous to the predefined pattern. However, PatScan was designed to detect general repeat not specific for CRISPRs, causing the inability of distinguishing the spacers and repeats in the whole CRISPR array. Later, several specific CRISPR identifiers came along, such as CRISPRFinder (19), PILER-CR (20), and CRT (21). The principle of CRISPRFinder (19) is using the suffix tree-based algorithm to find the maximal repeats that are clamped by the non-repeating sequences with similar length. Besides, PILER-CR (20) based on the alignment matrix identifies putative CRISPR arrays through searching local hits of the query genome to itself and uses sequence similarity, conservation, and length distribution to refine them. Different from CRISPRFinder and PILER-CR, CRT (21) does not rely on any central data structure but adopts the strategy of simple sequential scanning, which enables a high execution speed independent of the number of repeats in the given genome. Afterward, CRISPRDetect (22) based on k-mer and extension strategy was proposed and labeled itself with the improvement of utilizing the features of CRISPR loci especially mutations. CRISPRDetect (22) is more sensitive to short and degenerated repeats by scanning for the variant repeats under a low identity threshold in long spacers, but it incidentally brings the possibility of wrong segmentation of the large integral CRISPRs. The comparison of the advantages and disadvantages of the abovementioned basic CRISPRs identifiers was demonstrated in Table 1.

**Table 1:** The details of 5 basic tools for identifying CRISPR arrays.

**Tools**	**Language**	**Advantage**	**Disadvantage**	**Input**	**Output**	**Platform**	**Address**	**References**
PatScan	C++	1. Provide web server 2. Can be used to predict various genomic patterns	1. Cannot distinguish CRISPRs from other types of repeats 2. Require complex post-processing 3. Not fast when dealing with the large query set 4. The number of repeats requires predefined	DNA/protein sequences	Repeat sequences	Web server	https://patscan.seconDarymetabolites.org/	(18)
CRISPRFinder	Perl	1. Provide both reliable and questionable CRISPRs 2. Some predicted results can be directly retrieved from database CRISPRdb	1. Do not take repeat mutations into account 2. Behave poor in the detection of short or degenerate CRISPRs 3. Not fast when dealing with the large query set	DNA sequences	Repeat and spacer sequences	Web server	https://crispr.i2bc.paris-saclay.fr/Server/	(19)
PILER-CR	C++	1. Provide classification for CRISPRs 2. Can handle deletions and insertions in the repeats 3. Execute rapidly	1. Do not use the features to discriminate genuine CRISPRs 2. Cannot filter out tandem repeat sequences 3. Not user-friendly	DNA sequences	1. Repeat and spacer sequences 2. Cluster by similarity and position	Standalone program	http://www.drive5.com/pilercr/	(20)
CRT	Java	1. Speed is independent of the number of repeats 2. Relatively high reliability 3.Using simple data structure	1. Do not use the features to discriminate genuine CRISPRs 2. Behave poor in the detection of short or degenerate CRISPRs 3. Not user-friendly	DNA sequences	Repeat and spacer sequences	Standalone program	http://www.room220.com/crt/	(21)
CRISPRDetect	Perl	1. Provide additional information such as array direction and variations 2. Some predicted results can be directly retrieved from database CRISPRBank 3. Sensitive to short and degenerate arrays	1. Possibly mis-split larger integral CRISPRs into small arrays 2. Not fast when dealing with the large query set	DNA sequences or species name	1. Repeat and spacer sequences 2. Mutations 3. Potential Cas genes	Web server and Standalone program	http://crispr.otago.ac.nz/CRISPRDetect/	(22)

*Some tools derived from basic tools and tailored for specific purposes are introduced in main text and Table 2*.

Along with the diversity of research demand, there are some tools derived from the basic identifiers and tailored for different purposes (Table 2). One of the most popular purposes now is to explore the CRISPR diversity from metagenomic data and classify the CRISPR/Cas system. Due to the repetitive nature and population heterogeneity, it is hard to assemble CRISPRs from metagenomes using basic tools. Therefore, MinCED (23), MetaCRAST (24), Crass (25), and metaCRT (26) were developed. MinCED, Crass, and MetaCRT are all based on CRT (21) tool and implement the *de novo* detection. Moreover, MinCED and Crass have no need for prior knowledge of CRISPR arrays of which MinCED only detects spacers in reads without assembly and Crass assembles the reads into arrays. In contrast, metaCRT (26) integrates the reference-based and *de novo* detection. Besides, MetaCRAST (24), another reference-based method, searches for repeats pairing with the user-defined templates that could be identified by either other tools like CRISPRFinder, PILER-CR, and CRF or taxonomy, whereas its performance is inferior to Crass and MinCED for the poor taxonomic diversity. In addition, there are also some tools tailored for other purposes. For instance, if users want to compare the CRISPR arrays from different species, CRISPRcompar (27) comprising CRISPRcomparison and CRISPRtionary and basically derived from CRISPRFinder must be the best choice. Besides, CRF (28) based on CRT added random forest algorithm to make an extra filtration for invalid CRISPR arrays, but this learning-based tool may partially lose the ability to discover new CRISPRs. Beyond that, three representative tools are designed for CRISPR strand prediction using the characteristics of leader and repeat that include CRISPRstrand (29), CRISPRleader (31), and CRISPRDirection (37).

**Table 2:** The list of the tools and databases tailored for different purposes.

**Tool/Database**	**Purpose**	**Basic tool**	**Platform**	**Address**	**References**
MinCED	Explore CRISPR diversity from metagenome	CRT	Standalone program	https://github.com/ctSkennerton/minced	(23)
MetaCRAST	Explore CRISPR diversity from metagenome	CRT, PILER-CR, and CRISPRFinder	Standalone program	https://github.com/molleraj/MetaCRAST	(24)
Crass	Explore CRISPR diversity from metagenome	CRT	Standalone program	https://ctskennerton.github.io/crass/	(25)
metaCRT	Explore CRISPR diversity from metagenome	CRT	Standalone program	https://omics.informatics.indiana.edu/CRISPR/	(26)
CRISPRcompar	Compare CRISPRs from different species	CRISPRFinder	Web server	https://crispr.i2bc.paris-saclay.fr/CRISPRcompar/	(27)
CRF	Filter the invalid CRISPRs	CRT	Standalone program and web server	http://bioinfolab.miamioh.edu/crf/home.php	(28)
CRISPRstrand	Predict CRISPR orientation	CRT and CRISPRFinder	Web server	http://rna.informatik.uni-freiburg.de/CRISPRmap	(29)
CRISPRDirection	Predict CRISPR orientation	CRISPRDetect	Standalone program	http://bioanalysis.otago.ac.nz/CRISPRDirection/	(30)
CRISPRleader	Predict leader and CRISPR orientation	CRT	Standalone program	http://www.bioinf.uni-freiburg.de/Software/CRISPRleader/	(31)
CRISPRdisco	Classify CRISPR/Cas systems	MinCED (CRISPR identifier) BLAST (Cas detector)	Standalone program	https://github.com/CRISPRlab/CRISPRdisco	(32)
CRISPRCasFinder	Classify CRISPR/Cas systems	CRISPRFinder (CRISPR identifier) MacSyFinder (Cas detector)	Standalone program and web server	https://crisprcas.i2bc.paris-saclay.fr	(33)
CRISPRmap	Classify CRISPR/Cas systems	CRT, CRISPRFinder and CRISPRstrand (CRISPR identifier) HMMER (Cas detector)	Web server	http://rna.informatik.uni-freiburg.de/CRISPRmap	(34)
CRISPRone	Collect predicted CRISPRs, cas genes and false-CRISPRs	metaCRT (CRISPR identifier) HMMER (Cas detector)	Web server (database)	https://omics.informatics.indiana.edu/CRISPRone/	(35)
CRISPRCasdb	Collect predicted CRISPRs and cas genes	CRISPRCasFinder (CRISPR identifier) BLAST (Cas detector)	Web server (database)	https://crisprcas.i2bc.paris-saclay.fr/	(36)

### Incorporation With Cas Protein Detector

Other than the abovementioned tools only focusing on CRISPR arrays, recent tools integrate Cas protein detector to improve the classification capacity and enable the automated CRISPR/Cas system discovery. These tools determine the putative Cas protein by using the homologous sequence searcher such as BLAST (38) and HMMER (39), which compare the query Cas protein with the sequences in a known protein database. For example, CRISPRmap (34) is composed of CRT and CRISPRFinder for CRISPR array identification and HMMER for Cas protein annotation. CRISPRdisco (32) incorporates MinCED and BLAST to realize similar functions. Besides, CRISPRCasFinder based on CRISPRFinder for CRISPR array identification integrates the function of Cas protein detection by using a dedicated tool MacSyFinder (40), which is in essence HMMER. Except for the predictors, there are some databases collecting the predicted CRISPRs and Cas proteins such as CRISPRBank (30), CRISPRone (35), and CRISPRCasdb (CRISPRdb) (36).

Although much effort had been invested in the CRISPR/Cas system identification and classification, there are still some unsolved limitations. On one hand, identifying CRISPR arrays especially short arrays based only on pattern alignment or along with limited sequence information is not enough to accurately eliminate noises. It is an imperative trend, as the progression from basic tools to tailored tools, to excavate and incorporate more significant architectural and functional features such as the transcriptional polarity within CRISPRs (41) and regulatory relationships with endogenous genes in a bacterial host (42) to improve the prediction performance. On the other hand, current tools for Cas protein detection are majorly based on the annotation propagation by searching for homologous sequences, which narrows the possibility of discovering novel Cas proteins.

## Guide RNA Design and Assessment

As a key component of CRISPR/Cas system, gRNA specifies the target of Cas enzymes through PAM recognition. The quality of gRNA largely determines the efficacy and specificity of CRISPR/Cas-mediated editing. To date, there have been several types of RNAs found to play guiding roles via various mechanisms in different CRISPR/Cas systems (Figure 1), such as the mature crRNA in CRISPR/Cas12a (formerly Cpf1) system (43) and the hybrid of crRNA and tracRNA in CRISPR/Cas9 system (44). In this section, these RNAs with guiding functions have a joint name, gRNA.

With the wider applications of the CRISPR/Cas system, an increasing number of studies expressed their apprehensions over the incidental off-target effects, which may trigger the mis-editing at other loci and lead to unforeseeable phenotypic alterations (45, 46). Thereupon, designing an efficient and functional gRNA with both high on-target efficacy and low off-target mutations becomes the focus of much attention. Recent computational efforts have taken a massive step toward high-quality gRNA design. In what follows, we will set forth the usages and contributions of gRNA designers from two subsections, *Overview of the gRNA Designers* and *Special View Into Off-Target Activity*.

### Overview of the gRNA Designers

Owing to the simple architecture and superior operability, class 2 CRISPR/Cas systems (Figure 1) gain much wider applications. Consequently, almost all current *in silico* gRNA designers are developed for class 2 systems. The following description is also confined to the class 2 CRISPR systems.

**Figure 2:**
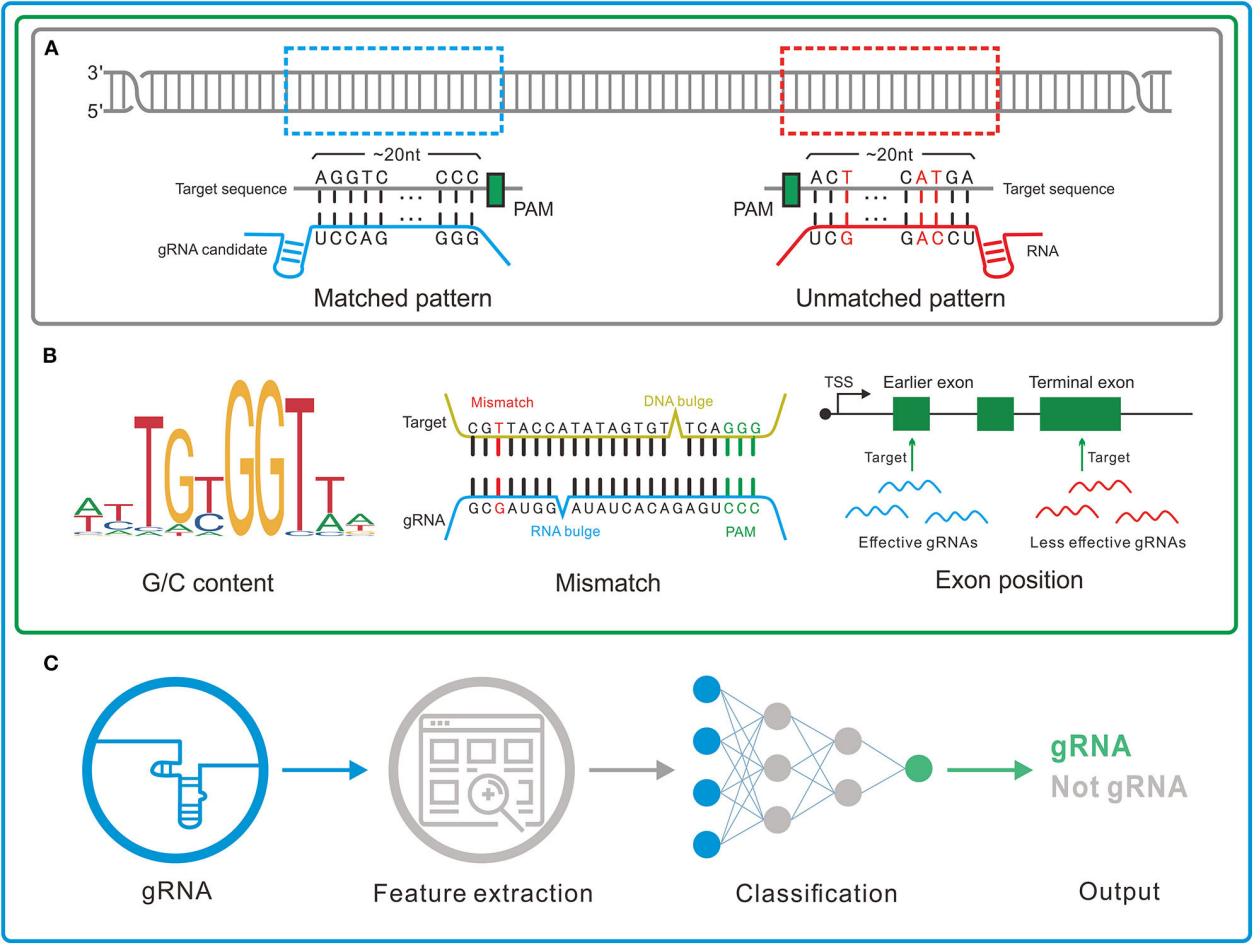
Three genres of guide RNA (gRNA) designers. **(A)** Pattern recognition genre. The tools in this genre depend on the base-pairing rule to determine the gRNAs. **(B)** Feature rule genre. A set of features such as G/C content, mismatch, and gRNA transcription method is used to filter out the unreliable or unconcerned gRNAs obtained by pattern recognition. **(C)** Machine learning genre. In this genre, machine learning algorithms are applied to integrate the effects of the features and thus more precisely identify the gRNAs.

By different inner principles, we divided the gRNA designers into three major genres (Figure 2). The characteristics of the representative tools in each genre were shown in Table 3.

**Table 3:** The details of 40 representative and commonly used gRNA designer.

**Tools**	**Cas effector**	**Target species**	**gRNA type^**$**^**	On-target method^*^	**Off-target prediction**	**PAM^**#**^**	**Scoring efficiency**	**CRISPRa/i**	**Platform**	**References**
Cas-OFFinder	Custom	Any species	S	PR	Yes	User-defined	No	No	Web server and Standalone program	(47)
SSFinder	Cas9	Any species	S	PR	No	NGG	No	No	Standalone program	(48)
CRISPRseek	Custom	Any species	S	PR	Yes	User-defined	No	No	Standalone program	(49)
flyCRISRP	Cas9	37 kinds of fly	S	PR	Yes	NGG/NRG	No	No	Web server	(50)
GT-Scan	Custom	105 kinds of vertebrate, invertebrate and plant	S	PR	Yes	User-defined	No	No	Web server	(51)
CasFinder	Cas9	Any species	S	PR	Yes	User-defined	No	No	Standalone program	(52)
Breaking-Cas	Custom	All eukaryotic genomes available in ENSEMBL	S	PR	Yes	User-defined	Yes	No	Web server	(53)
Crisflash	Custom	Any species	S	PR	Yes	User-defined	Yes	No	Standalone program	(54)
sgRNACas9	Cas9	Any species	S	FR	Yes	NGG	No	No	Standalone program	(55)
CRISPRdirect	Custom	671 kinds of vertebrate, invertebrate and plant	S	FR	Yes	User-defined	No	No	Web server	(30)
Cas-Designer	Custom	Any species	S	FR	Yes	User-defined	No	No	Web server and Standalone program	(56)
CT-Finder	Cas9	17 kinds of vertebrate, invertebrate and plant	S	FR	Yes	User-defined	No	No	Web server	(57)
CGAT	Cas9	6 kinds of plants	S	FR	Yes	NGG	No	No	Web server	(58)
CROP-IT	Cas9	Human and mouse	S	FR	Yes	NGG/NNG	Yes	No	Web server	(59)
CRISPR-ERA	Cas9	9 kinds of vertebrate and invertebrate	S	FR	Yes	NGG/NAG/NRG	Yes	Yes	Web server	(60)
CRISPR-RT	Cas13a	Any species	S	FR	Yes	User-defined	No	No	Web server and Standalone program	(61)
CRISPR multitargeter	Custom	12 kinds of vertebrate, invertebrate and plant	S	FR	Yes	User-defined	Yes	No	Web server	(62)
SSC	Cas9	Human and mouse	S	ML	Yes	NGG	No	Yes	Web server and Standalone program	(63)
EuPaGDT	Custom	Any species	S	ML	Yes	User-defined	Yes	No	Web server	(64)
E-CRISP	Custom	55 kinds of vertebrate, invertebrate and plant	P	ML	Yes	User-defined	Yes	Yes	Web server	(65)
Crispr-P 2.0	Cas9, Cas12a	49 kinds of plants	S	ML	Yes	NGG/NRG/NNAGAAW/NNNNGMTT/NNGRRT/TTTV/ TTN/YCN/CCW/YYC/AWG/CC/MMA/NG	Yes	No	Web server	(66)
CCTop (CRISPRater)	Cas9, Cas12a	Any species	S	ML	Yes	NGG/NRG/NGA/NGCG/TTTN/YTN/NNGRRT/NNNRRT/ NNNNGATT/NNAGAAW/NAAAAC/NNNNRYAC	Yes	No	Web server and Standalone program	(67, 68)
CRISPR-offinder	Custom	Any species	S	ML	Yes	User-defined	Yes	No	Standalone program	(69)
CRISPETa	Cas9	Any species	P	ML	Yes	NGG	Yes	No	Web server and Standalone program	(70)
CHOPCHOP v3	Cas9, Cas12a, Cas13	Any species	S	ML	Yes	User-defined	Yes	Yes	Web server and Standalone program	(71)
CRISPR-DT	Cas9, Cas12a	15 kinds of vertebrate, invertebrate and plant	S	ML	Yes	User-defined	Yes	No	Web server	(72)
pgRNAFinder	Custom	10 kinds of vertebrate and invertebrate	P	ML	Yes	User-defined	Yes	No	Web server and Standalone program	(73)
WU-CRISPR	Cas9	Any species but better in human and mouse	S	ML	Yes	NGG	Yes	No	Web server and Standalone program	(74)
CRISPRscan	Cas9, Cas12a	24 kinds of vertebrate and invertebrate	S	ML	Yes	NGG/TTTN/TTTV	Yes	No	Web server	(75)
sgRNA Scorer v2.0	Custom	Any species	S	ML	Yes	User-defined	Yes	No	Web server and Standalone program	(76)
TUSCAN	Cas9	105 kinds of vertebrate, invertebrate and plant	S	ML	Yes	User-defined	Yes	No	Web server	(77)
GPP (Azimuth)	Cas9	Human, mouse and rat	S	ML	Yes	NGG/NNGRR	Yes	Yes	Web server	(78)
CRISPOR	Custom	Any species	S	ML	Yes	User-defined	Yes	No	Web server and Standalone program	(79)
DeepCpf1	Cas12a	Human	S	ML	No	TTTG	Yes	No	Web server and Standalone program	(80)
DeepCas9	Cas9	Human	S	ML	No	NGG	Yes	No	Standalone program	(81)
GuideScan	Cas9, Cas12a	6 kinds of vertebrate and invertebrate	P	ML	Yes	NGG/TTTN	Yes	No	Web server	(82)
CLD	Custom	Any species	S	ML	Yes	User-defined	Yes	No	Standalone program	(83)
CRISPR-Local	Custom	Any species	S	ML	Yes	User-defined	Yes	No	Web server and Standalone program	(84)
PAVOOC	Cas9	Human and mouse	S	ML	Yes	NGG/NAG/	Yes	No	Web server	(85)
DeepCRISPR	Cas9	Human	S	ML	Yes	NGG	Yes	No	Web server	(86)

1. Some tools have both web and standalone versions, and some parameters like species and PAM in web version are limited but in standalone version are not. Here we only show the less strict setting.

2. The information of each tool in this table refers to the newest version rather than the old version in publication.

$ gRNA type: S, single gRNA; P, paired gRNA.

* On-target method: PR, pattern reorganization; FR, feature rule; ML, machine learning.

# *PAM: W = A/T; R = A/G; M = A/C; V = C/G/A; Y = C/T*.

**1) Pattern recognition genre** (Figure 2A) relying on base-pairing principle. In this category, tools search for a piece of sequence comprising a short PAM and around 20-bp candidate gRNA complementary to the query sequence in a specified genome. The fewer mismatches the candidate gRNA has, the greater on-target possibility it likely produces. Besides, the specific PAM should be predefined for its diversity in different CRISPR/Cas systems. Another factor influencing gRNA pattern is the transcription methods, in which U6 and T7 promoters, respectively, require G and GG at 5'end of gRNA (87, 88). Some tools such as CRISPRseek (49) and flyCRISPR (50) take it into account while others such as SSFinder (48) and GT-Scan (51) do not. Besides, for individual studies, Crisflash (54) is able to improve the accuracy by incorporating user-supplied somatic mutation data into pattern matching.

**2) Feature rule genre** (Figure 2B). The subsequent finding that editing activities vary across different target sites indicates the inherent disparity of some targets in the sensitivity to cleavage (89–92) and thus ushers a series of explorations to seek out the key features that influence the targeting efficacy (93, 94). These features include G/C content of gRNAs (high or low G/C content indicates less activity) (95), frequency of frameshift mutations (negative with CRISPR efficacy) (96), poly-T sequences (a typical terminator for gRNA transcription) (97, 98), compositions of nucleobases involved in Cas binding preference (the presence of PAM-preceding G and the absence of pyrimidines in the last 4nt of gRNA spacers are preferred) (63), exon position (lower efficacy when gRNAs targeting the terminal coding exon rather than the earlier exons) (99), the status of the motif- and feature-enriched ~10–12 nt proximal to PAM in spacer sequences dubbed seed region (associated with pairing process) (100, 101), and so on. Tools in this genre always integrate several measurable features with the basic pattern recognition approach to provide more information about candidate gRNAs and target sites. According to feature indexes and the corresponding thresholds, users can lay down their own rules to filter out the gRNAs with poor reliability or of no interest. For instance, Cas-Designer (56) lists putative gRNAs along with G/C proportions and out-of-frame scores that indicate the frequency of in-frame mutations. Besides, CRISPR-ERA (60) constructs a simple scoring rule by arbitrarily quantifying and weighting the information of G/C content, poly-T motifs and target locations.

Tools affiliated to this genre provide separate assessment or arbitrary combinations for multiple features rather than perform an integrative analysis on their interactive contributions, which may perplex users about how to balance the probably discordant results of multiple features. Machine learning algorithms found an exit for this dilemma.

**3) Machine learning genre** (Figure 2C). Given that the weights of multiple features remain uncertain, researchers resort to mathematical algorithms that systematically integrate features for refining optimal gRNA. These models always differ in algorithms and information in training data. For example, Doench et al. (95) (Rule set 1) observed the depletion rates of gRNAs targeting cell surface markers in mouse and human cells and attributed them to the intrinsic nucleotide composition of target sequences, which then acted as training data to construct the logistic regression classifier for gRNA activity prediction. Moreover, combining the changes in expression of cell surface markers (Rule set 1) (95) and drug resistance pathways (Rule set 2), Azimuth (102) trained by the information of not only nucleotide composition but also secondary structure of gRNAs and the relative location of target sites to the transcription start site (TSS) shows improved performance. Unlike above methods using phenotypic changes to measure activity, some others relying on mutations detected by sequencing were proposed. CRISPRscan (75), a linear regression model, investigated the effect of nucleotide composition on CRISPR/Cas9 efficacy by taking the gRNA-induced mutation rates of target sequences in zebrafish embryos as the signal of activity. In addition, sgRNA Scorers v2.0 (76) based on the support vector machine used similar training data from sequencing (mutation rates of the targets in human HEK293T cells). Likewise, TUSCAN (77) reanalyzed the published data and improved the prediction performance by adding the features of flanking target regions and replacing the algorithm with random forest. For fear of the potential biases caused by the manual selection of features in abovementioned tools based on the conventional machine learning algorithm, up-to-date tools (80, 81, 86) based on deep learning algorithm minimize the biases by automating feature extraction of which DeepCRISPR (86) is particularly noteworthy for unifying both on-target and off-target predictions into one framework and additionally allowing for epigenetic features despite using phenotype-driven data.

Phenotype-driven models are largely influenced by the target positions, some of which far from TSS less likely trigger phenotypic change and would be misclassified into the negative. In contrast, sequencing-based models implement more direct measurement of genetic mutations and have consequently superior generalizability (77). In a word, phenotype-driven models get the upper hand when users are more interested in the functional outcome of gRNA-induced mutations, while sequencing-based models occupy wider application fields if only genotype alterations are focused.

Even though *in silico* gRNA designers experience a positive evolution, the performances of machine learning-based tools remain difficult to maintain due to the varying features across different species and Cas enzymes requiring an exclusive loading process. Therefore, users were recommended to use the tools based on feature rules if their data are not eligible for the machine learning algorithm. Except for the abovementioned categorical characteristic, gRNA designers also have other distinguishable specialties such as the one-step customization of paired gRNA (pgRNA) for large fragment deletion [e.g., CRISPETa (70), pgRNAFinder (73), and GuideScan (82)], special consideration for CRISPR activation or interference (CRISPRa/i) (103) [e.g., SSC (63), CRISPR-ERA (60), and CHOPCHOP v3.0 (71)], application platform, off-target prediction, and so on. These specialties endow the tools with distinctive ability in particular fields and thus give users more choices for their specific purpose. Moreover, some commercial tools should also be helpful for their visual interface, online consultation, and one-stop ordering service, such as Synthego (https://www.synthego.com/products/bioinformatics/crispr-design-tool) based on the Azimuth algorithm (102) and IDT (https://www.idtdna.com/site/order/designtool/index/CRISPR_CUSTOM) based on their own evaluation algorithm, but most of the commercial tools were designed for the most popular CRISPR/Cas9 system and provided less support for other types of CRISPR systems. Table 3 recording the detailed comparison of some commonly used gRNA designers provides a more brief reference. Since no tool can be omnipotent, the pre-conditions and anticipated purpose should be fully thought before the gRNA designer selection.

### Special View Into Off-Target Activity

Off-target activity leading to mis-editing on the unintended regions had been widely reported, which can trigger unpredictably adverse outcomes (104, 105). Undoubtedly, experimental methods including whole-genome sequencing [e.g., CIRCLE-seq (106), GUIDE-seq (107), DISCOVER-seq (108), Digenome-seq (109), BLESS (110), and HTGTS (111)] and the improved VIVO strategy (112) are relatively robust and accurate for off-target identification. Nonetheless, the labor- and cost-intensive sequencing methods are not affordable for every researcher and sometimes unnecessary, thus urging the coming and progress of *in silico* methods.

The most typical and convenient *in silico* strategy for off-target risk evaluation is to align the short gRNA sequences sometimes with PAMs to reference genome to detect mismatch number and position by repurposing the alignment tools [e.g., Bowtie (113), PatMaN (114), and BWA (115)], which is exemplified by GT-Scan (51), CRISPR-RT (61), E-CRISP (65), and so on. However, short read aligners likely induce a large proportion of false-negative errors due to their maximum allowable mismatches. When mismatch number exceeds 2 in a certain read, the accuracy of aligners gets a drastic decline (116). The comparison between the gold standard GUIDE-seq (107) and the alignment strategy revealed that numerous high-mismatch off-targets and even one-mismatch off-targets cannot be detected by only alignment (107). On the other hand, the limited mismatches are hard to represent the authentic off-targets and may cause false-positives. This is supported by an experiment based on SITE-seq, which found that the alignment-based off-targets largely outnumbered the validated off-targets by up to 10-fold (117).

**Figure 3:**
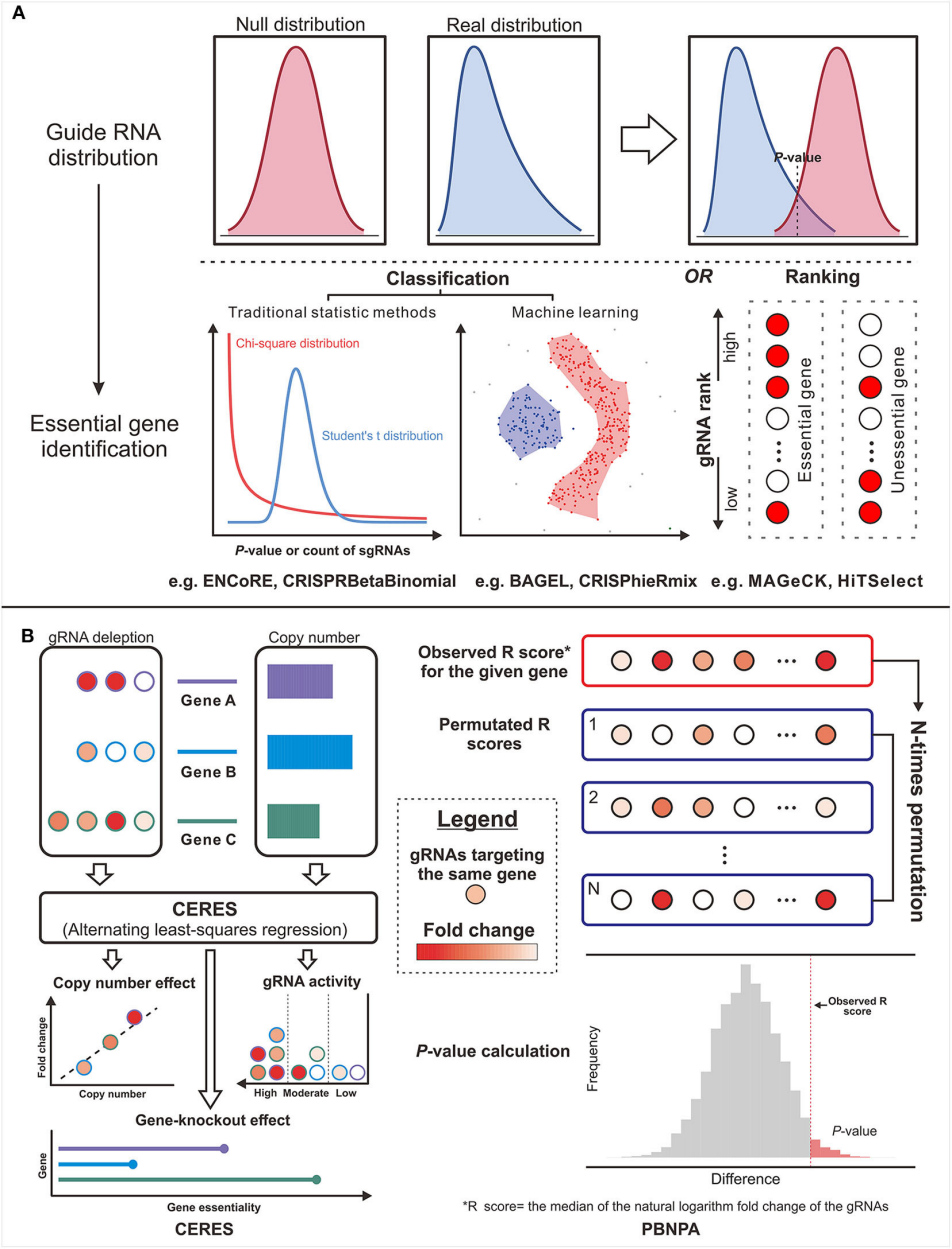
The design concepts of the gene essentiality evaluators. **(A)** The typical genre is from the guide RNA (gRNA) distribution comparison to essential gene identification. **(B)** Two methods in untypical ways: The left panel illustrates the workflow of CERES, which corrects the copy number effect based on the alternating least-squares regression. The right panel illustrates the workflow of PBNPA based on the permutation test.

Aiming to narrow both types of errors and realize the quantitative evaluation on off-target possibility, some features and scoring systems are incorporated into the prediction programs (Figure 3). For example, CCTop (67) and CROP-IT (59), respectively, incorporate seed region and DNase-sensitive region with mismatch number to grade the potential off-target sites using handcraft rules. Furthermore, mismatches with a few extra bases (DNA bulge) or missing bases (RNA bulge) in target sequences were once reported to be tolerable (118). COSMID (119) lists the number of bulges rather than incorporates it into the scoring rule for the lack of experimentally validated data. Despite the additional features in the above tools, the off-target searching method they used still relies on alignment strategy, which is not as reliable as the sequencing-based off-target source used in following tools. By introducing the mutated gRNAs into cells and measuring the gRNA abundance to quantify the off-target activities, CFD (102) exhibited more dominant power and has been widely repurposed in other tools such as CRISPR-Local (84), GuideScan (82), and GPP sgRNA designer (78). In contrast with the discontinued MIT-Broad algorithm (120) whose scans area confines to 20-bp sequences, CFD (102) covers PAM as it found non-canonical PAMs tend to induce potential off-target events (102). Subsequently, researchers proved CFD's superior performance by comparison with experimental data (121). However, it should be noted that CFD only aggregates the off-targets within a certain gene rather than a genome-wide scale.

To overcome the drawbacks of handcraft rules and extend the aggregation scale, recent developers are more inclined to machine learning algorithm (Figure 3). CRISTA (122) constructed a random forest model based on the enlarged feature set covering mismatch types (wobble and bulge), chromatin accessibility, DNA enthalpy, and DNA geometry. Regrettably, the complex feature set creates a double-edge sword, which indeed enhances the prediction performance but also restricts the application scope. Using simpler features, Elevation (123), a genome-wide aggregation model based on Naive Bayes, provides a more systematic assessment for multi-loci off-target detection. Besides, the state-of-the-art deep learning algorithm was also applied using only sequencing data and achieved a relatively better result (124). Deep learning takes more full advantage of experimental datasets, whereas the lack of aggregation function and the narrow feature set remain an intractable limitation. The evolution of the original off-target scoring systems is illustrated in Figure 3.

In conclusion, an optimal gRNA should possess not only maximum on-target efficacy but also minimum off-target activity, which requires *in silico* designers equipped with both high accuracy and robustness. Moreover, the incorporation of more functional features is a key to improve prediction performance. As genetic researches are stepping forward, some additional factors such as histone modification (93, 125) and Cas protein variants (126) were found to exert significant influences on editing efficacy and specificity. Besides, what wins the most attention recently must be individual variance that was reported to be discriminately associated with the genesis or destruction of the potential off-target activity (127–129). Therefore, the applications of CRISPR/Cas system especially for clinical purposes would better be specified into the individual scale to control the risk of deleterious side effects.

## Post-Experimental Assistance

CRISPR/Cas-mediated high-throughput screening has become a main force to impute phenotypic changes to large-scale genetic or epigenetic alterations. In screening, the pooled gRNA library is amplified, packaged, and transfected into the host cells (130, 131). The transfected cells are screened for a phenotype of interest, of which the survived would be sequenced to measure gRNA abundance. After that, the major challenges turn to be how to precisely transform the differential gRNA abundances after selection to the gene essentiality evaluation and how to systematically enumerate and visualize the CRISPR/Cas-induced mutations. Bioinformaticians have provided innovative solutions using computational methods to boost the experimental procedure as shown in Figure 1B. Hereinafter, *in silico* methods are introduced in three parts: *Essential Gene Identification, Decipherment of the CRISPR-Induced Mutations*, and *Database for Experimental Data Collection*.

### Essential Gene Identification

**Figure 4:**
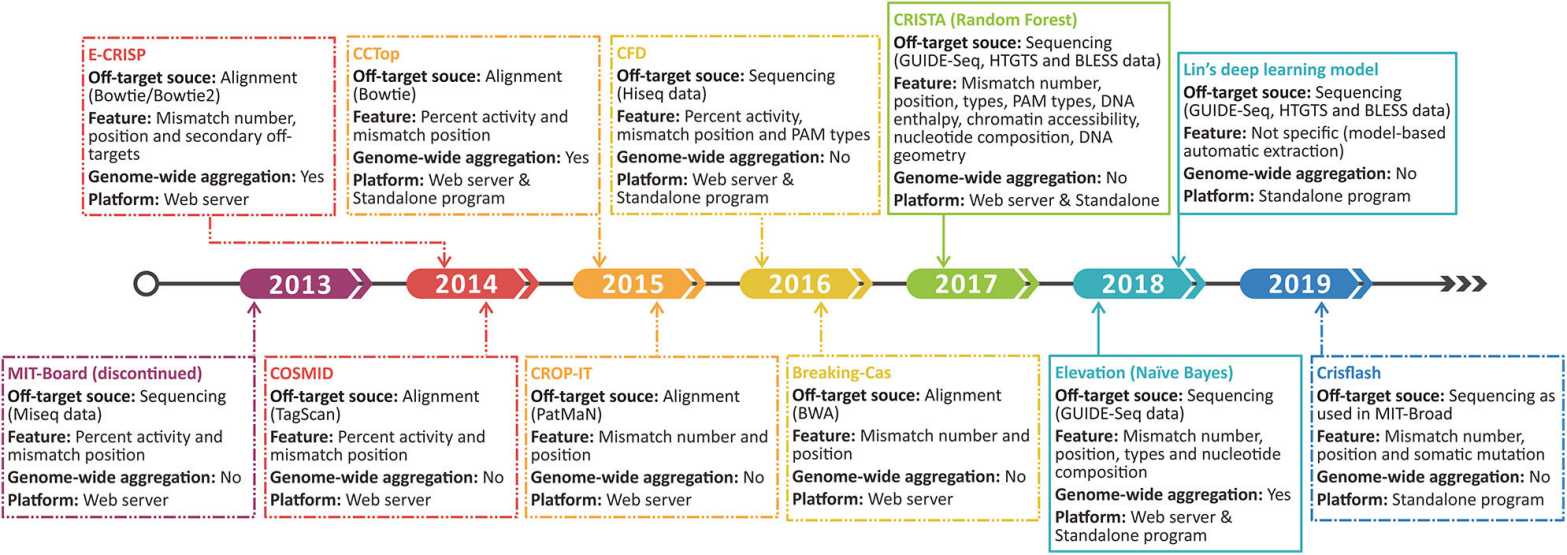
Time line shows the development progress of the original off-target scoring system. The dashed and sealed boxes represent the handcraft and machine learning-based scoring systems, respectively.

Since CRISPR/Cas-mediated screening strategy was proposed, several sorts of approaches have been put forward to estimate gene essentiality. At the early stage, some off-the-shelf tools for RNA-seq expression analysis [e.g., edgeR (132), baySeq (133), and DEseq2 (134)] served as makeshifts for CRISPR studies. The algorithms designed for RNA interference (RNAi) screens [e.g., RIGER (135) and RSA (136)] were also regarded as substitutes. However, these algorithms cannot exactly achieve satisfying suitability for CRISPR screens due to various deficiencies including the lack of quality control, unrobustness to variable gRNA coverage per gene, and the weak power in controlling the bias toward small sample size or gRNAs with small read count. To fill the gaps, some dedicated methods have been emerging constantly (Figure 4, Table 4). The typical strategy (Figure 4) is to compare the read count distribution of gRNA with control and then aggregate the variances of multiple gRNAs with the same target into an estimate of gene-level effect.

**Table 4:** The details of the gene essentiality evaluators for CRISPR screens.

**Tools**	**gRNA-level**	**Gene-level**	Functions^*^				**Interface**	**References**
	**model**	**model**	**QC**	**HI**	**VI**	**FI**		
MAGeCKFlute	Negative binomial distribution	RRA/MLE	Yes	Yes	Yes	Yes	Command line	(137)
HiTSelect	Poisson distribution	Stochastic multi-objective ranking method	No	Yes	Yes	Yes	Command line and Graphic interface (standalone)	(138)
ScreenBEAM	Gaussian distribution	Bayesian hierarchical model	No	Yes	No	No	Command line	(139)
BAGEL	Training distribution	Bayesian classifier	No	Yes	No	No	Command line	(140)
ENCoRE	Gaussian distribution	Student's *t*-test	Yes	Yes	Yes	No	Graphic interface (standalone)	(141)
PBNPA	–	Non-parametric permutation	No	Yes	No	No	Command line	(142)
JACKS	Gaussian distribution	Empirical Bayesian model	No	Yes	No	No	Command line	(143)
CERES	-	Alternating least squares	No	Yes	No	No	Command line	(144)
CRISPhieRmix	Hierarchical mixture distribution	Expectation maximization algorithm	No	Yes	No	No	Command line	(145)
CRISPRBetaBinomial	Beta-binomial distribution	Fisher's method	No	Yes	Yes	No	Command line and Graphic interface (web)	(146)

* *Functions: QC, quality control; HI, hit identification; VI, visualization; FI, functional inference*.

MAGeCK-RRA (147) based on the negative binomial model and robust rank aggregation (RRA) is the first tool customized for prioritizing gRNAs, performing gene-level ranking and identifying the enriched pathways. To extend the functions, MAGeCK-RRA (147) was further updated to scMAGeCK (148) for single-cell CRISPR screening (a novel technique combining pooled CRISPR screening with single-cell RNA-seq, which enables the identification of gRNAs at single-cell resolution from sequencing by modifying the lentiviral vector) and MAGeCKFlute (137) with optional ranking algorithm (maximum likelihood estimation) (149), gRNA outlier removal by network essentiality scoring tool (150), and various accessory functions including upstream quality control and downstream visualization. For some novices without programming expertise, command-line programs are hard to tame and the graphical workflow, ENCoRE (141), seems more user-friendly, whereas the rough processing of gene ranking may induce unreliable results. Likewise, a universal analyzer, HiTSelect (138), is designed for both RNAi and CRISPR screens, whereas Poisson distribution used to fit the active gRNA abundance is not applicable because the mean and variance of gRNA count are always not equal. Considering that the variance of gRNA count can be either smaller or greater than the mean, Jeong et al. (146) developed CRISPRBetaBinomial based on beta-binomial distribution model and gained the superior sensitivity as well as lower false-negative rate as expected. Totally different in gene-level statistic, BAGEL (140) and JACKs (143) used the reference sets composed of the identified essential and non-essential genes to analyze the query data. Even though these prior knowledge-based methods reward excellent performance, the required compatibility between reference and query sets and the prohibitive update of the pre-set data remain the critical handicaps for popularization. Allowing for the varying effects of gRNAs targeting the same gene especially in CRISPRa/i screens, CRISPhieRmix (145) took a hierarchical mixture model to deconvolute the gRNA distribution and calculate a posterior probability for genes, in which sufficient gRNAs per gene are required to ensure the full discovery of essential genes.

Other than the above methods affiliated to typical strategy, the methods in other ways provide more options for particular problems. For example, CERES (144) incorporated copy number effect and thus realized improved specificity in the realm of cancer cells (the left panel of Figure 4). Furthermore, PBNPA (142) (the right panel of Figure 4) permuted gRNA labels to compute gene-level *p*-values, which may outperform the competitors when encountering the small amounts of gRNAs per gene or low sequencing depth. Similarly, ScreenBEAM (139) is another skillful solution for low-quality data owing to the direct estimation on the gene level. The characteristics of existing essentiality evaluators are listed in Table 4.

In general, despite leaving copy number effect out of consideration, MAGeCK (137) remains the most widely used tool in various biological fields such as identifying cancer drivers (151), drug targets (152), and pathway components (153). Its prominent advantages over other tools are the all-around service covering both upstream and downstream analyses, relative ease of use, and the excellent ranking criteria that deal well with variable gRNA efficacies. Meanwhile, there are still positions for other tools when facing the cases they are adept at. ScreenBEAM (139) for low-quality data and ENCoRE (141) for novice users are two representative examples.

### Decipherment of the CRISPR-Induced Mutations

Owing to the outstanding feasibility and versatility, type II CRISPR/Cas9 and type V CRISPR/Cas12a occupy the most dominant position in practical use. Double-strand breaks (DSBs) created by Cas9 or Cas12a cleavage can be repaired via several kinds of pathways, which induce the mixed mutations. The repair pathways mainly include (1) non-homologous end joining (NHEJ) (154), which is an error-prone repair pathway and may induce random insertions and deletions (INDELs); (2) homology-directed repair (HDR) (155), which relies on a donor template homologous to the sequence around DSB site to realize the precise editing or correction; and (3) microhomology-mediated end-joining (MMEJ) (156), where the single-stranded overhangs generated by the nuclease are annealed at the microhomologies (typically 5–25 bp) existing both upstream and downstream of DSB. Then, two major methods were used to dissect the mutational outcome. First, some machine learning-based tools, such as in Delphi (157), FORECasT (158), and Lindel (159), used the characteristic of sequence context to achieve a great prediction on the distribution of mutations. However, as similar as other learning-based tools, the application of these tools was largely subject to the training set and cannot be spread across different CRISPR systems and species. Secondly, next-generation sequencing (NGS) can not only detect the mutations but also classify the mutation types and mutagenesis efficiency. Nonetheless, transforming millions of sequencing signals to quantitative and comparable data remains challenging and needs mathematical aids from *in silico* tools. The fundamental workflow of these tools is similar to the standard high-throughput sequencing analysis including quality control, trimming adaptor, alignment, and quantification. The main difference in the existing tools will be demonstrated as follows.

1) Alignment strategy. The existing tools adopt either local alignment to the reference amplicons [e.g., CRIS.py (160), CRISPR-DAV (161), and CRISPR-GA (162)] or global alignment to an entire reference genome [e.g., CrispRVariants (163) and AmpliconDIVider (164)]. The local strategy is apt to miscount the candidate off-target reads, while global strategy makes it difficult to quantitatively deconvolute the mixed outcomes of gene editing. Besides, some tools [e.g., CRISPResso2 (165) and BATCH-GE (166)] combine both strategies by predefining cut sites. Collectively, choosing an alignment strategy depends on what kind of information users prefer.

2) Deconvolution of the mixed mutations. As mentioned above, three major pathways (NHEJ, MMEJ, and HDR) jointly participate in DSB repair. In contrast to the unpredictable mutations generated by NHEJ, precise modifications generated by HDR and MMEJ are preferred for purposive gene editing. Therefore, classifying the modified alleles is essential for determining the mutant sites and mutagenesis efficiency. The tools adopting local strategy [e.g., CRISPResso2 (165), CRIS.py (160), CRISPR-DAV (161), and CRISPR-GA (162)] align reads to the expected HDR amplicon and the reference amplicon and then identify the modification status by the comparisons of alignment rates and sequence identities. Moreover, some tools [e.g., ampliconDIVider (164), CRIS.py (160), and CRISPResso2 (165)] enable the quantification of in-frame occurrences and potential splice sites according to mutation location and sequence length. The mutations located in the coding region with relatively conserved length are always regarded as in-frame, while the others are frame-shift. Yet regrettably, the tool for distinguishing MMEJ-induced mutations remains unavailable.

3) Applicability for base editors. For fear of the random introduction of INDELs in canonical CRISPR/Cas experiments, base editors, the fusions composed of a catalytically impaired Cas enzyme to a base deaminase that operates on single strand, can directly install point mutations by mediating base conversion without DSB generation (167, 168). Conventional tools only for INDEL quantification cannot detect the varying combinations of base conversion induced by the base editor. Interestingly, CRIS.py (160) and CRISPResso2 (165) compensate for this vacancy through searching the pre-set nucleotide substitution rule.

Additionally, whether the tools are equipped with visualization and the execution platform is worth considering. The detailed information of existing CRISPR NGS data analyzers is listed in Table 5.

**Table 5:** The details of the existing CRISPR NGS data analyzers.

**Tools**	**Alignment**		Mutation quantification^*^			**Visualization**	**Platform**	**References**
	**Strategy**	**Mapper**	**MD**	**FQ**	**BE**			
CRISPR-GA	Local	BLAT	Yes	No	No	No	Web server and Standalone	(162)
CRISPR-DAV	Global and Local	BWA and ABRA	Yes	No	No	Yes	Standalone	(161)
BATCH-GE	Global and Local	BWA MEM	Yes	No	No	Yes	Standalone	(166)
CrispRVariants	Global	BWA MEM	No	Yes	No	Yes	Standalone	(163)
Cas-analyzer	Local	EMBOSS Needle	Yes	No	No	No	Web server	(169)
CRISPRmatch	Local	BWA	No	No	No	Yes	Standalone	(170)
AmpliconDIVider	Global	NovoAlign	No	Yes	No	No	Standalone	(164)
CRIS.py	Local	Text based alignment^#^	Yes	Yes	Yes	No	Standalone	(160)
CRISPResso2	Global and Local	EMBOSS Needle	Yes	Yes	Yes	Yes	Web server and Standalone	(165)

* Mutation quantification: MD, mutation deconvolution; FQ, frameshift quantification; BE, base editor.

# *Text-based alignment is an author-defined method*.

### Database for Experimental Data Collection

The applications of CRISPR/Cas screening massively expand in gene function exploration, so does the need for the open databases for validated data collection where researchers can easily get access to raw or processed data. To satisfy the urgent need, several repositories had been built (Table 6). Of note, compared with the databases only recording results but without any comparisons of screening results among different researches [e.g., CRISPRz (171), CrisprGE (172), CRISPRlnc (151), and BioGRID ORCS (176)], GenomeCRISPR (173) based on 84 high-throughput screens additionally provides the intuitive comparisons of gRNA efficacies as well as perturbation phenotypes under specific conditions. Instead of collecting the gRNA information, PICKLES (174) reanalyzed the raw screening data and compared the essentiality of a certain gene across multiple experiments, tissues, or cells. Another two independent databases tailored for human cancer research are Sanger DepMap (175) and Broad DepMap (144), which record the information of gene dependencies in cancer cell lines through analyzing the CRISPR/Cas9 screening data.

**Table 6:** The list of 7 existing databases collecting the CRISPR screening data.

**Database**	**Species**	**CRISPR type**	Gene type^*^	**Gene count^**#**^**	**Last update**	**Address**	**References**
CRISPRz	Zebrafish	CRISPRko	PCG	610	March 2016	https://research.nhgri.nih.gov/CRISPRz/	(171)
CrisprGE	32 kinds of vertebrate, invertebrate and plant	CRISPRko/ki	PCG, miRNA	223	June 2015	http://crdd.osdd.net/servers/crisprge/	(172)
CRISPRlnc	8 kinds of vertebrate, invertebrate and plant	CRISPRko, CRISPRa/i	LncRNA	304	September 2019	http://www.crisprlnc.org/	(151)
		base editor					
GenomeCRISPR	Human	CRISPRko, CRISPRa/i	PCG, lncRNA, miRNA	28,655	November 2017	http://genomecrispr.dkfz.de/	(173)
PICKLES	Human	CRISPRko/ki, CRISPRa/i	PCG	20,953	2019	https://hartlab.shinyapps.io/pickles/	(174)
Sanger DepMap	Human	CRISPRko	PCG	18,009	April 2019	https://score.depmap.sanger.ac.uk/	(175)
Broad DepMap	Human	CRISPRko	PCG	18,333	December 2019	https://depmap.org/portal/	(144)
BioGRID ORCS	*Drosophila*, human and mouse	CRISPRko, CRISPRa/i	PCG, lncRNA, miRNA	58,161	July 2019	https://orcs.thebiogrid.org/	(176)

* Gene type: PCG, protein-coding gene; lncRNA, long noncoding RNA; miRNA, micro RNA.

# *Gene count is across all species the database involved in rather than for a single one*.

Furthermore, there are some databases [e.g., Anti-CRISPRdb (177) and CRISPRminer (178)] recording the anti-CRISPR proteins in phage that had been experimentally validated to inhibit the activity of CRISPR/Cas system and reduce off-target events (179).

## Conclusion and Perspective

CRISPR/Cas systems have navigated researchers to traverse through the dark where they are left flat-footed by the complex functional annotation. However, the advances in experimental techniques still cannot promise CRISPR/Cas system an effortless and expedite manner, which, therefore, needs essential assistance from *in silico* methods. Our study makes a comprehensive summary and comparisons on the released tools from two perspectives: pre-experimental guidance (CRISPR/Cas system identification and gRNA design) and post-experimental analysis (gene essentiality evaluation, decipherment of the experimental outcome, and data collection). The characteristics of tools based on different design principles and frameworks had been elucidated hereinbefore, which hopefully guide users to make more reasonable choices for their specific data and purposes.

Unfortunately, CRISPR/Cas system cannot yet reach a satisfying achievement in practical use. Current strategies for technical improvement mainly probe into two aspects. On one hand, the most reliable and effective approach is to optimize the experimental technique, which is well-exemplified by the fusion of catalytically impaired Cas enzymes to other engineered proteins for constructing the riskless systems such as CRISPRa/i (103), base editor (167), and prime editor (180) and enhancing the efficiency of precise repair (181). Yet experimental improvement cannot cover all facets, let alone guarantee affordable cost. At that time, *in silico* tools, the second aspect, are of importance even if there is still a long way ahead such as how chromatin environment affects the on-target and off-target activities, whether the effects are fixed or varying across tissue and organisms, how to solve the disparity of training set in machine learning-based tools that may cause the poor versatility, and how to combine the individual information into the personalized gRNA design. To the best of our knowledge, the hypotheses of tool optimization are: (1) For CRISPR/Cas system identification, precisely distinguishing CRISPR arrays from other similar repeats requires the incorporation of more distinct features such as the interactions with other genes in the host (42) and the intra-genus conservation (41); (2) For gRNA design, except feature expansion and algorithm optimization, the individual variance associated with on-target and off-target activities (127–129) should be taken into account. Current tools such as Crisflash (54) and CRISPR-Local (84) considering only somatic mutation are far from satisfactory. It is envisioned that *in silico* tools covering more individual characteristics such as chromatin environment, accessibility, and exon expression promise more reliable prediction, especially for the clinical purpose; (3) For gene essentiality evaluation, existing tools are not as all-powerful as we expected, which misinterpret the uncertain relationships between the mean and variance of gRNA count, neglect the copy number effect, or lack accessory functions; (4) For the deconvolution of mutations, combination of microhomology predictor and local alignment to reference may pave a new way for quantifying the MMEJ-induced mutations.

The urgent demand for optimizing *in silico* methods cannot mask the truth that they have made tremendous contributions to biological researches. It is increasingly expected that the progress in computational methods will push CRISPR/Cas system into a higher stage and even assist in an earlier realization of clinical popularization.

## Author Contributions

All authors listed have made a substantial, direct and intellectual contribution to the work, and approved it for publication.

## Conflict of Interest

The authors declare that the research was conducted in the absence of any commercial or financial relationships that could be construed as a potential conflict of interest.

## References

[1] Gupta SK Shukla P. Gene editing for cell engineering: trends and applications. Crit Rev Biotechnol. (2017) 37:672–84. 10.1080/07388551.2016.1214557 27535623

[2] Knott GJ Doudna JA. CRISPR-Cas guides the future of genetic engineering. Science. (2018) 361:866–9. 10.1126/science.aat5011 30166482 PMC6455913

[3] Barrangou R Fremaux C Deveau H Richards M Boyaval P Moineau S. CRISPR provides acquired resistance against viruses in prokaryotes. Science. (2007) 315:1709–12. 10.1126/science.1138140 17379808

[4] Leenay RT Beisel CL. Deciphering, communicating, and engineering the CRISPR PAM. J Mol Biol. (2017) 429:177–91. 10.1016/j.jmb.2016.11.024 27916599 PMC5235977

[5] Pickar-Oliver A Gersbach CA. The next generation of CRISPR-Cas technologies and applications. Nat Rev Mol Cell Biol. (2019) 20:490–507. 10.1038/s41580-019-0131-5 31147612 PMC7079207

[6] Koonin EV Makarova KS Zhang F. Diversity, classification and evolution of CRISPR-Cas systems. Curr Opin Microbiol. (2017) 37:67–78. 10.1016/j.mib.2017.05.008 28605718 PMC5776717

[7] Shmakov S Smargon A Scott D Cox D Pyzocha N Yan W. Diversity and evolution of class 2 CRISPR-Cas systems. Nat Rev Microbiol. (2017) 15:169–82. 10.1038/nrmicro.2016.184 28111461 PMC5851899

[8] Tang Y Fu Y. Class 2 CRISPR/Cas: an expanding biotechnology toolbox for and beyond genome editing. Cell Biosci. (2018) 8:59. 10.1186/s13578-018-0255-x 30459943 PMC6233275

[9] Zhu S Li W Liu J Chen CH Liao Q Xu P. Genome-scale deletion screening of human long non-coding RNAs using a paired-guide RNA CRISPR-Cas9 library. Nat Biotechnol. (2016) 34:1279–86. 10.1038/nbt.3715 27798563 PMC5592164

[10] Xu L Wang J Liu Y Xie L Su B Mou D. CRISPR-edited stem cells in a patient with HIV and acute lymphocytic leukemia. N Engl J Med. (2019) 381:1240–7. 10.1056/NEJMoa1817426 31509667

[11] Yin H Xue W Anderson DG. CRISPR-Cas: a tool for cancer research and therapeutics. Nat Rev Clin Oncol. (2019) 16:281–95. 10.1038/s41571-019-0166-8 30664678

[12] Ran FA Hsu PD Wright J Agarwala V Scott DA Zhang F Genome engineering using the CRISPR-Cas9 system. Nat Protoc. (2013) 8:2281–308. 10.1038/nprot.2013.143 24157548 PMC3969860

[13] Li B Zeng C Dong Y. Design and assessment of engineered CRISPR-Cpf1 and its use for genome editing. Nat Protoc. (2018) 13:899–914. 10.1038/nprot.2018.004 29622802 PMC6383568

[14] Strecker J Jones S Koopal B Schmid-Burgk J Zetsche B Gao L. Engineering of CRISPR-Cas12b for human genome editing. Nat Commun. (2019) 10:212. 10.1038/s41467-018-08224-4 30670702 PMC6342934

[15] Ozcan A Pausch P Linden A Wulf A Schuhle K Heider J. Type IV CRISPR RNA processing and effector complex formation in aromatoleum aromaticum. Nat Microbiol. (2019) 4:89–96. 10.1038/s41564-018-0274-8 30397343

[16] Brouns SJ Jore MM Lundgren M Westra ER Slijkhuis RJ Snijders AP. Small CRISPR RNAs guide antiviral defense in prokaryotes. Science. (2008) 321:960–4. 10.1126/science.1159689 18703739 PMC5898235

[17] Marraffini LA Sontheimer EJ. CRISPR interference limits horizontal gene transfer in staphylococci by targeting DNA. Science. (2008) 322:1843–5. 10.1126/science.1165771 19095942 PMC2695655

[18] Dsouza M Larsen N Overbeek R. Searching for patterns in genomic data. Trends Genet. (1997) 13:497–8. 10.1016/S0168-9525(97)01347-4 9433140

[19] Grissa I Vergnaud G Pourcel C. CRISPRFinder: a web tool to identify clustered regularly interspaced short palindromic repeats. Nucleic Acids Res. (2007) 35:W52–57. 10.1093/nar/gkm360 17537822 PMC1933234

[20] Edgar RC. PILER-CR: fast and accurate identification of CRISPR repeats. BMC Bioinformatics. (2007) 8:18. 10.1186/1471-2105-8-18 17239253 PMC1790904

[21] Bland C Ramsey TL Sabree F Lowe M Brown K Kyrpides NC. CRISPR recognition tool (CRT): a tool for automatic detection of clustered regularly interspaced palindromic repeats. BMC Bioinformatics. (2007) 8:209. 10.1186/1471-2105-8-209 17577412 PMC1924867

[22] Biswas A Staals RH Morales SE Fineran PC Brown CM. CRISPRDetect: a flexible algorithm to define CRISPR arrays. BMC Genomics. (2016) 17:356. 10.1186/s12864-016-2627-0 27184979 PMC4869251

[23] Skennerton C. T (2016). MinCED: Mining CRISPRs in Environmental Datasets. Available online at: https://github.com/ctSkennerton/minced/tree/master (accessed September 16, 2020).

[24] Moller AG Liang C. MetaCRAST: reference-guided extraction of CRISPR spacers from unassembled metagenomes. PeerJ. (2017) 5:e3788. 10.7717/peerj.3788 28894651 PMC5592083

[25] Skennerton CT Imelfort M Tyson GW. Crass: identification and reconstruction of CRISPR from unassembled metagenomic data. Nucleic Acids Res. (2013) 41:e105. 10.1093/nar/gkt183 23511966 PMC3664793

[26] Rho M Wu YW Tang H Doak TG Ye Y. Diverse CRISPRs evolving in human microbiomes. PLoS Genet. (2012) 8:e1002441. 10.1371/journal.pgen.1002441 22719260 PMC3374615

[27] Grissa I Vergnaud G Pourcel C. CRISPRcompar: a website to compare clustered regularly interspaced short palindromic repeats. Nucleic Acids Res. (2008) 36:W145–48. 10.1093/nar/gkn228 18442988 PMC2447796

[28] Wang K Liang C. CRF: detection of CRISPR arrays using random forest. PeerJ. (2017) 5:e3219. 10.7717/peerj.3219 28462029 PMC5407274

[29] Alkhnbashi OS Costa F Shah SA Garrett RA Saunders SJ Backofen R. CRISPRstrand: predicting repeat orientations to determine the crRNA-encoding strand at CRISPR loci. Bioinformatics. (2014) 30:i489–96. 10.1093/bioinformatics/btu459 25161238 PMC4147912

[30] Naito Y Hino K Bono H Ui-Tei K. CRISPRdirect: software for designing CRISPR/Cas guide RNA with reduced off-target sites. Bioinformatics. (2015) 31:1120–3. 10.1093/bioinformatics/btu743 25414360 PMC4382898

[31] Alkhnbashi OS Shah SA Garrett RA Saunders SJ Costa F Backofen R. Characterizing leader sequences of CRISPR loci. Bioinformatics. (2016) 32:i576–85. 10.1093/bioinformatics/btw454 27587677

[32] Crawley AB Henriksen JR Barrangou R. CRISPRdisco: an automated pipeline for the discovery and analysis of CRISPR-Cas systems. CRISPR J. (2018) 1:171–81. 10.1089/crispr.2017.0022 31021201 PMC6636876

[33] Couvin D Bernheim A Toffano-Nioche C Touchon M Michalik J Neron B. CRISPRCasFinder, an update of CRISRFinder, includes a portable version, enhanced performance and integrates search for Cas proteins. Nucleic Acids Res. (2018) 46:W246–51. 10.1093/nar/gky425 29790974 PMC6030898

[34] Lange SJ Alkhnbashi OS Rose D Will S Backofen R. CRISPRmap: an automated classification of repeat conservation in prokaryotic adaptive immune systems. Nucleic Acids Res. (2013) 41:8034–44. 10.1093/nar/gkt606 23863837 PMC3783184

[35] Zhang Q Ye Y. Not all predicted CRISPR-Cas systems are equal: isolated cas genes and classes of CRISPR like elements. BMC Bioinformatics. (2017) 18:92. 10.1186/s12859-017-1512-4 28166719 PMC5294841

[36] Pourcel C Touchon M Villeriot N Vernadet JP Couvin D Toffano-Nioche C. CRISPRCasdb a successor of CRISPRdb containing CRISPR arrays and cas genes from complete genome sequences, and tools to download and query lists of repeats and spacers. Nucleic Acids Res. (2019) 48:D535–44. 10.1093/nar/gkz915 31624845 PMC7145573

[37] Biswas A Fineran PC Brown CM. Accurate computational prediction of the transcribed strand of CRISPR non-coding RNAs. Bioinformatics. (2014) 30:1805–13. 10.1093/bioinformatics/btu114 24578404

[38] Camacho C Coulouris G Avagyan V Ma N Papadopoulos J Bealer K. BLAST+: architecture and applications. BMC Bioinformatics. (2009) 10:421. 10.1186/1471-2105-10-421 20003500 PMC2803857

[39] Eddy SR. Accelerated profile HMM searches. PLoS Comput Biol. (2011) 7:e1002195. 10.1371/journal.pcbi.1002195 22039361 PMC3197634

[40] Abby SS Neron B Menager H Touchon M Rocha EP. MacSyFinder: a program to mine genomes for molecular systems with an application to CRISPR-Cas systems. PLoS ONE. (2014) 9:e110726. 10.1371/journal.pone.0110726 25330359 PMC4201578

[41] Bernick DL Cox CL Dennis PP Lowe TM. Comparative genomic and transcriptional analyses of CRISPR systems across the genus pyrobaculum. Front Microbiol. (2012) 3:251. 10.3389/fmicb.2012.00251 22811677 PMC3396285

[42] Chen J Li T Zhou X Cheng L Huo Y Zou J. Characterization of the clustered regularly interspaced short palindromic repeats sites in *Streptococcus mutans* isolated from early childhood caries patients. Arch Oral Biol. (2017) 83:174–80. 10.1016/j.archoralbio.2017.07.023 28783550

[43] Zetsche B Gootenberg JS Abudayyeh OO Slaymaker IM Makarova KS Essletzbichler P. Cpf1 is a single RNA-guided endonuclease of a class 2 CRISPR-Cas system. Cell. (2015) 163:759–71. 10.1016/j.cell.2015.09.038 26422227 PMC4638220

[44] Karvelis T Gasiunas G Miksys A Barrangou R Horvath P Siksnys V. crRNA and tracrRNA guide Cas9-mediated DNA interference in *Streptococcus thermophilus*. RNA Biol. (2013) 10:841–51. 10.4161/rna.24203 23535272 PMC3737341

[45] Schaefer KA Wu WH Colgan DF Tsang SH Bassuk AG Mahajan VB. Unexpected mutations after CRISPR-Cas9 editing *in vivo*. Nat Methods. (2017) 14:547–8. 10.1038/nmeth.4293 28557981 PMC5796662

[46] Anderson KR Haeussler M Watanabe C Janakiraman V Lund J Modrusan Z. CRISPR off-target analysis in genetically engineered rats and mice. Nat Methods. (2018) 15:512–4. 10.1038/s41592-018-0011-5 29786090 PMC6558654

[47] Bae S Park J Kim JS. Cas-OFFinder: a fast and versatile algorithm that searches for potential off-target sites of Cas9 RNA-guided endonucleases. Bioinformatics. (2014) 30:1473–5. 10.1093/bioinformatics/btu048 24463181 PMC4016707

[48] Upadhyay SK Sharma S. SSFinder: high throughput CRISPR-Cas target sites prediction tool. Biomed Res Int. (2014) 2014:742482. 10.1155/2014/742482 25089276 PMC4095993

[49] Zhu LJ Holmes BR Aronin N Brodsky MH. CRISPRseek: a bioconductor package to identify target-specific guide RNAs for CRISPR-Cas9 genome-editing systems. PLoS ONE. (2014) 9:e108424. 10.1371/journal.pone.0108424 25247697 PMC4172692

[50] Gratz SJ Cummings AM Nguyen JN Hamm DC Donohue LK Harrison MM. Genome engineering of *Drosophila* with the CRISPR RNA-guided Cas9 nuclease. Genetics. (2013) 194:1029–35. 10.1534/genetics.113.152710 23709638 PMC3730909

[51] O'brien A Bailey TL. GT-scan: identifying unique genomic targets. Bioinformatics. (2014) 30:2673–5. 10.1093/bioinformatics/btu354 24860161 PMC4155256

[52] Aach J Mali P Church GM CasFinder: flexible algorithm for identifying specific Cas9 targets in genomes. bioRxiv. [Preprint]. (2014) 005074. 10.1101/005074

[53] Oliveros JC Franch M Tabas-Madrid D San-Leon D Montoliu L Cubas P. Breaking-Cas-interactive design of guide RNAs for CRISPR-Cas experiments for ENSEMBL genomes. Nucleic Acids Res. (2016) 44:W267–71. 10.1093/nar/gkw407 27166368 PMC4987939

[54] Jacquin ALS Odom DT Lukk M. Crisflash: open-source software to generate CRISPR guide RNAs against genomes annotated with individual variation. Bioinformatics. (2019) 35:3146–7. 10.1093/bioinformatics/btz019 30649181 PMC6735888

[55] Xie S Shen B Zhang C Huang X Zhang Y sgRNAcas9: a software package for designing CRISPR sgRNA and evaluating potential off-target cleavage sites. PLoS ONE. (2014) 9:e100448. 10.1371/journal.pone.0100448 PMC4067335 24956386

[56] Park J Bae S Kim JS. Cas-Designer: a web-based tool for choice of CRISPR-Cas9 target sites. Bioinformatics. (2015) 31:4014–6. 10.1093/bioinformatics/btv537 26358729

[57] Zhu H Misel L Graham M Robinson ML Liang C. CT-Finder: a web service for CRISPR optimal target prediction and visualization. Sci Rep. (2016) 6:25516. 10.1038/srep25516 27210050 PMC4876460

[58] Brazelton VA Jr Zarecor S Wright DA Wang Y Liu J Chen K. A quick guide to CRISPR sgRNA design tools. GM Crops Food. (2015) 6:266–76. 10.1080/21645698.2015.1137690 26745836 PMC5033207

[59] Singh R Kuscu C Quinlan A Qi Y Adli M. Cas9-chromatin binding information enables more accurate CRISPR off-target prediction. Nucleic Acids Res. (2015) 43:e118. 10.1093/nar/gkv575 26032770 PMC4605288

[60] Liu H Wei Z Dominguez A Li Y Wang X Qi LS. CRISPR-ERA: a comprehensive design tool for CRISPR-mediated gene editing, repression and activation. Bioinformatics. (2015) 31:3676–8. 10.1093/bioinformatics/btv423 26209430 PMC4757951

[61] Zhu H Richmond E Liang C. CRISPR-RT: a web application for designing CRISPR-C2c2 crRNA with improved target specificity. Bioinformatics. (2018) 34:117–9. 10.1093/bioinformatics/btx580 28968770

[62] Prykhozhij SV Rajan V Gaston D Berman JN CRISPR multitargeter: a web tool to find common and unique CRISPR single guide RNA targets in a set of similar sequences. PLoS ONE. (2015) 10:e0119372. 10.1371/journal.pone.0119372 PMC4351176 25742428

[63] Xu H Xiao T Chen CH Li W Meyer CA Wu Q. Sequence determinants of improved CRISPR sgRNA design. Genome Res. (2015) 25:1147–57. 10.1101/gr.191452.115 26063738 PMC4509999

[64] Peng D Tarleton R. EuPaGDT: a web tool tailored to design CRISPR guide RNAs for eukaryotic pathogens. Microb Genom. (2015) 1:e000033. 10.1099/mgen.0.000033 28348817 PMC5320623

[65] Heigwer F Kerr G Boutros M. E-CRISP: fast CRISPR target site identification. Nat Methods. (2014) 11:122–3. 10.1038/nmeth.2812 24481216

[66] Liu H Ding Y Zhou Y Jin W Xie K Chen LL. CRISPR-P 2.0: an improved CRISPR-Cas9 tool for genome editing in plants. Mol Plant. (2017) 10:530–32. 10.1016/j.molp.2017.01.003 28089950

[67] Stemmer M Thumberger T Del Sol Keyer M Wittbrodt J Mateo JL CCTop: an intuitive, flexible and reliable CRISPR/Cas9 target prediction tool. PLoS ONE. (2015) 10:e0124633. 10.1371/journal.pone.0124633 PMC4409221 25909470

[68] Labuhn M Adams FF Ng M Knoess S Schambach A Charpentier EM. Refined sgRNA efficacy prediction improves large- and small-scale CRISPR-Cas9 applications. Nucleic Acids Res. (2018) 46:1375–85. 10.1093/nar/gkx1268 29267886 PMC5814880

[69] Zhao C Zheng X Qu W Li G Li X Miao YL. CRISPR-offinder: a CRISPR guide RNA design and off-target searching tool for user-defined protospacer adjacent motif. Int J Biol Sci. (2017) 13:1470–8. 10.7150/ijbs.21312 29230095 PMC5723913

[70] Pulido-Quetglas C Aparicio-Prat E Arnan C Polidori T Hermoso T Palumbo E. Scalable design of paired CRISPR guide RNAs for genomic deletion. PLoS Comput Biol. (2017) 13:e1005341. 10.1371/journal.pcbi.1005341 28253259 PMC5333799

[71] Labun K Montague TG Krause M Torres Cleuren YN Tjeldnes H Valen E. CHOPCHOP v3: expanding the CRISPR web toolbox beyond genome editing. Nucleic Acids Res. (2019) 47:W171–4. 10.1093/nar/gkz365 31106371 PMC6602426

[72] Zhu H Liang C. CRISPR-DT: designing gRNAs for the CRISPR-Cpf1 system with improved target efficiency and specificity. Bioinformatics. (2019) 35:2783–9. 10.1093/bioinformatics/bty1061 30615056

[73] Xiong Y Xie X Wang Y Ma W Liang P Songyang Z. pgRNAFinder: a web-based tool to design distance independent paired-gRNA. Bioinformatics. (2017) 33:3642–4. 10.1093/bioinformatics/btx472 28961776 PMC5870604

[74] Wong N Liu W Wang X. WU-CRISPR: characteristics of functional guide RNAs for the CRISPR/Cas9 system. Genome Biol. (2015) 16:218. 10.1186/s13059-015-0784-0 26521937 PMC4629399

[75] Moreno-Mateos MA Vejnar CE Beaudoin JD Fernandez JP Mis EK Khokha MK. CRISPRscan: designing highly efficient sgRNAs for CRISPR-Cas9 targeting *in vivo*. Nat Methods. (2015) 12:982–8. 10.1038/nmeth.3543 26322839 PMC4589495

[76] Chari R Yeo NC Chavez A Church GM. sgRNA Scorer 2.0: a species-independent model to predict CRISPR/Cas9 activity. ACS Synth Biol. (2017) 6:902–4. 10.1021/acssynbio.6b00343 28146356 PMC5793212

[77] Wilson LOW Reti D O'brien AR Dunne RA Bauer DC. High activity target-site identification using phenotypic independent CRISPR-Cas9 core functionality. CRISPR J. (2018) 1:182–190. 10.1089/crispr.2017.0021 31021206

[78] Sanson KR Hanna RE Hegde M Donovan KF Strand C Sullender ME. Optimized libraries for CRISPR-Cas9 genetic screens with multiple modalities. Nat Commun. (2018) 9:5416. 10.1038/s41467-018-07901-8 30575746 PMC6303322

[79] Concordet JP Haeussler M. CRISPOR: intuitive guide selection for CRISPR/Cas9 genome editing experiments and screens. Nucleic Acids Res. (2018) 46:W242–5. 10.1093/nar/gky354 29762716 PMC6030908

[80] Kim HK Min S Song M Jung S Choi JW Kim Y. Deep learning improves prediction of CRISPR-Cpf1 guide RNA activity. Nat Biotechnol. (2018) 36:239–41. 10.1038/nbt.4061 29431740

[81] Kim HK Kim Y Lee S Min S Bae JY Choi JW. SpCas9 activity prediction by DeepCas9, a deep learning-based model with unparalleled generalization performance. bioRxiv. [Preprint]. (2019) 636472. 10.1101/636472 31723604 PMC6834390

[82] Perez AR Pritykin Y Vidigal JA Chhangawala S Zamparo L Leslie CS. GuideScan software for improved single and paired CRISPR guide RNA design. Nat Biotechnol. (2017) 35:347–9. 10.1038/nbt.3804 28263296 PMC5607865

[83] Heigwer F Zhan T Breinig M Winter J Brugemann D Leible S. CRISPR library designer (CLD): software for multispecies design of single guide RNA libraries. Genome Biol. (2016) 17:55. 10.1186/s13059-016-0915-2 27013184 PMC4807595

[84] Sun J Liu H Liu J Cheng S Peng Y Zhang Q. CRISPR-Local: a local single-guide RNA (sgRNA) design tool for non-reference plant genomes. Bioinformatics. (2019) 35:2501–3. 10.1093/bioinformatics/bty970 30500879

[85] Schaefer M Clevert DA Weiss B Steffen A. PAVOOC: designing CRISPR sgRNAs using 3D protein structures and functional domain annotations. Bioinformatics. (2019) 35:2309–10. 10.1093/bioinformatics/bty935 30445568 PMC6596878

[86] Chuai G Ma H Yan J Chen M Hong N Xue D. DeepCRISPR: optimized CRISPR guide RNA design by deep learning. Genome Biol. (2018) 19:80. 10.1186/s13059-018-1459-4 29945655 PMC6020378

[87] Sander JD Joung JK. CRISPR-Cas systems for editing, regulating and targeting genomes. Nat Biotechnol. (2014) 32:347–55. 10.1038/nbt.2842 24584096 PMC4022601

[88] Zhang T Gao Y Wang R Zhao Y. Production of guide RNAs *in vitro* and *in vivo* for CRISPR using ribozymes and RNA polymerase II promoters. Bio Protoc. (2017) 7:e2148. 10.21769/BioProtoc.2148 28603751 PMC5463609

[89] Fu Y Foden JA Khayter C Maeder ML Reyon D Joung JK. High-frequency off-target mutagenesis induced by CRISPR-Cas nucleases in human cells. Nat Biotechnol. (2013) 31:822–6. 10.1038/nbt.2623 23792628 PMC3773023

[90] Fu Y Sander JD Reyon D Cascio VM Joung JK. Improving CRISPR-Cas nuclease specificity using truncated guide RNAs. Nat Biotechnol. (2014) 32:279–84. 10.1038/nbt.2808 24463574 PMC3988262

[91] Koike-Yusa H Li Y Tan EP Velasco-Herrera Mdel C Yusa K. Genome-wide recessive genetic screening in mammalian cells with a lentiviral CRISPR-guide RNA library. Nat Biotechnol. (2014) 32:267–73. 10.1038/nbt.2800 24535568

[92] Shalem O Sanjana NE Hartenian E Shi X Scott DA Mikkelson T. Genome-scale CRISPR-Cas9 knockout screening in human cells. Science. (2014) 343:84–7. 10.1126/science.1247005 24336571 PMC4089965

[93] Chari R Mali P Moosburner M Church GM. Unraveling CRISPR-Cas9 genome engineering parameters via a library-on-library approach. Nat Methods. (2015) 12:823–6. 10.1038/nmeth.3473 26167643 PMC5292764

[94] Horlbeck MA Witkowsky LB Guglielmi B Replogle JM Gilbert LA Villalta JE. Nucleosomes impede Cas9 access to DNA *in vivo* and *in vitro*. Elife. (2016) 5:e12677. 10.7554/eLife.12677.022 26987018 PMC4861601

[95] Doench JG Hartenian E Graham DB Tothova Z Hegde M Smith I. Rational design of highly active sgRNAs for CRISPR-Cas9-mediated gene inactivation. Nat Biotechnol. (2014) 32:1262–7. 10.1038/nbt.3026 25184501 PMC4262738

[96] Bae S Kweon J Kim HS Kim JS. Microhomology-based choice of Cas9 nuclease target sites. Nat Methods. (2014) 11:705–6. 10.1038/nmeth.3015 24972169

[97] Billon P Bryant EE Joseph SA Nambiar TS Hayward SB Rothstein R. CRISPR-mediated base editing enables efficient disruption of eukaryotic genes through induction of STOP codons. Mol Cell. (2017) 67:1068–79.e1064. 10.1016/j.molcel.2017.08.008 28890334 PMC5610906

[98] Tong Y Whitford CM Robertsen HL Blin K Jorgensen TS Klitgaard AK. Highly efficient DSB-free base editing for streptomycetes with CRISPR-BEST. Proc Natl Acad Sci USA. (2019) 116:20366–75. 10.1073/pnas.1913493116 31548381 PMC6789908

[99] Wang T Wei JJ Sabatini DM Lander ES. Genetic screens in human cells using the CRISPR-Cas9 system. Science. (2014) 343:80–4. 10.1126/science.1246981 24336569 PMC3972032

[100] Jiang F Doudna JA. CRISPR-Cas9 structures and mechanisms. Annu Rev Biophys. (2017) 46:505–29. 10.1146/annurev-biophys-062215-010822 28375731

[101] Graf R Li X Chu VT Rajewsky K. sgRNA sequence motifs blocking efficient CRISPR/Cas9-mediated gene editing. Cell Rep. (2019) 26:1098–103.e1093. 10.1016/j.celrep.2019.01.024 30699341 PMC6352712

[102] Doench JG Fusi N Sullender M Hegde M Vaimberg EW Donovan KF. Optimized sgRNA design to maximize activity and minimize off-target effects of CRISPR-Cas9. Nat Biotechnol. (2016) 34:184–91. 10.1038/nbt.3437 26780180 PMC4744125

[103] Gilbert LA Horlbeck MA Adamson B Villalta JE Chen Y Whitehead EH. Genome-scale CRISPR-mediated control of gene repression and activation. Cell. (2014) 159:647–61. 10.1016/j.cell.2014.09.029 25307932 PMC4253859

[104] Alkan F Wenzel A Anthon C Havgaard JH Gorodkin J. CRISPR-Cas9 off-targeting assessment with nucleic acid duplex energy parameters. Genome Biol. (2018) 19:177. 10.1186/s13059-018-1534-x 30367669 PMC6203265

[105] Hajiahmadi Z Movahedi A Wei H Li D Orooji Y Ruan H. Strategies to increase on-target and reduce off-target effects of the CRISPR/Cas9 system in plants. Int J Mol Sci. (2019) 20:3719. 10.3390/ijms20153719 31366028 PMC6696359

[106] Tsai SQ Nguyen NT Malagon-Lopez J Topkar VV Aryee MJ Joung JK. CIRCLE-seq: a highly sensitive *in vitro* screen for genome-wide CRISPR-Cas9 nuclease off-targets. Nat Methods. (2017) 14:607–14. 10.1038/nmeth.4278 28459458 PMC5924695

[107] Tsai SQ Zheng Z Nguyen NT Liebers M Topkar VV Thapar V. GUIDE-seq enables genome-wide profiling of off-target cleavage by CRISPR-Cas nucleases. Nat Biotechnol. (2015) 33:187–97. 10.1038/nbt.3117 25513782 PMC4320685

[108] Wienert B Wyman SK Richardson CD Yeh CD Akcakaya P Porritt MJ. Unbiased detection of CRISPR off-targets *in vivo* using DISCOVER-Seq. Science. (2019) 364:286–9. 10.1101/469635 31000663 PMC6589096

[109] Kim D Bae S Park J Kim E Kim S Yu HR. Digenome-seq: genome-wide profiling of CRISPR-Cas9 off-target effects in human cells. Nat Methods. (2015) 12:237–43. 10.1038/nmeth.3284 25664545

[110] Ran FA Cong L Yan WX Scott DA Gootenberg JS Kriz AJ. *In vivo* genome editing using *Staphylococcus aureus* Cas9. Nature. (2015) 520:186–91. 10.1038/nature14299 25830891 PMC4393360

[111] Frock RL Hu J Meyers RM Ho YJ Kii E Alt FW Genome-wide detection of DNA double-stranded breaks induced by engineered nucleases. Nat Biotechnol. (2015) 33:179–86. 10.1038/nbt.3101 25503383 PMC4320661

[112] Akcakaya P Bobbin ML Guo JA Malagon-Lopez J Clement K Garcia SP. *In vivo* CRISPR editing with no detectable genome-wide off-target mutations. Nature. (2018) 561:416–9. 10.1038/s41586-018-0500-9 30209390 PMC6194229

[113] Langmead B Trapnell C Pop M Salzberg SL. Ultrafast and memory-efficient alignment of short DNA sequences to the human genome. Genome Biol. (2009) 10:R25. 10.1186/gb-2009-10-3-r25 19261174 PMC2690996

[114] Prufer K Stenzel U Dannemann M Green RE Lachmann M Kelso J. PatMaN: rapid alignment of short sequences to large databases. Bioinformatics. (2008) 24:1530–1. 10.1093/bioinformatics/btn223 18467344 PMC2718670

[115] Li H Durbin R. Fast and accurate short read alignment with burrows-wheeler transform. Bioinformatics. (2009) 25:1754–60. 10.1093/bioinformatics/btp324 19451168 PMC2705234

[116] Hoffmann S Otto C Kurtz S Sharma CM Khaitovich P Vogel J. Fast mapping of short sequences with mismatches, insertions and deletions using index structures. PLoS Comput Biol. (2009) 5:e1000502. 10.1371/journal.pcbi.1000502 19750212 PMC2730575

[117] Cameron P Fuller CK Donohoue PD Jones BN Thompson MS Carter MM. Mapping the genomic landscape of CRISPR-Cas9 cleavage. Nat Methods. (2017) 14:600–6. 10.1038/nmeth.4284 28459459

[118] Lin Y Cradick TJ Brown MT Deshmukh H Ranjan P Sarode N CRISPR/Cas9 systems have off-target activity with insertions or deletions between target DNA and guide RNA sequences. Nucleic Acids Res. (2014) 42:7473–85. 10.1093/nar/gku402 24838573 PMC4066799

[119] Cradick TJ Qiu P Lee CM Fine EJ Bao G. COSMID: a web-based tool for identifying and validating CRISPR/cas off-target sites. Mol Ther Nucleic Acids. (2014) 3:e214. 10.1038/mtna.2014.64 25462530 PMC4272406

[120] Hsu PD Scott DA Weinstein JA Ran FA Konermann S Agarwala V. DNA targeting specificity of RNA-guided Cas9 nucleases. Nat Biotechnol. (2013) 31:827–32. 10.1038/nbt.2647 23873081 PMC3969858

[121] Haeussler M Schonig K Eckert H Eschstruth A Mianne J Renaud JB. Evaluation of off-target and on-target scoring algorithms and integration into the guide RNA selection tool CRISPOR. Genome Biol. (2016) 17:148. 10.1186/s13059-016-1012-2 27380939 PMC4934014

[122] Abadi S Yan WX Amar D Mayrose I. A machine learning approach for predicting CRISPR-Cas9 cleavage efficiencies and patterns underlying its mechanism of action. PLoS Comput Biol. (2017) 13:e1005807. 10.1371/journal.pcbi.1005807 29036168 PMC5658169

[123] Listgarten J Weinstein M Kleinstiver BP Sousa AA Joung JK Crawford J. Prediction of off-target activities for the end-to-end design of CRISPR guide RNAs. Nat Biomed Eng. (2018) 2:38–47. 10.1038/s41551-017-0178-6 29998038 PMC6037314

[124] Lin J Wong KC. Off-target predictions in CRISPR-Cas9 gene editing using deep learning. Bioinformatics. (2018) 34:i656–63. 10.1093/bioinformatics/bty554 30423072 PMC6129261

[125] Uusi-Makela MIE Barker HR Bauerlein CA Hakkinen T Nykter M Ramet M. Chromatin accessibility is associated with CRISPR-Cas9 efficiency in the zebrafish (Danio rerio). PLoS ONE. (2018) 13:e0196238. 10.1371/journal.pone.0196238 29684067 PMC5912780

[126] Choi GCG Zhou P Yuen CTL Chan BKC Xu F Bao S Combinatorial mutagenesis en masse optimizes the genome editing activities of SpCas9. Nat Methods. (2019) 16:722–30. 10.1038/s41592-019-0473-0 31308554

[127] Canver MC Lessard S Pinello L Wu Y Ilboudo Y Stern EN. Variant-aware saturating mutagenesis using multiple Cas9 nucleases identifies regulatory elements at trait-associated loci. Nat Genet. (2017) 49:625–34. 10.1038/ng.3793 28218758 PMC5374001

[128] Lessard S Francioli L Alfoldi J Tardif JC Ellinor PT Macarthur DG. Human genetic variation alters CRISPR-Cas9 on- and off-targeting specificity at therapeutically implicated loci. Proc Natl Acad Sci USA. (2017) 114:E11257–66. 10.1073/pnas.1714640114 29229813 PMC5748207

[129] Liu G Yin K Zhang Q Gao C Qiu JL. Modulating chromatin accessibility by transactivation and targeting proximal dsgRNAs enhances Cas9 editing efficiency *in vivo*. Genome Biol. (2019) 20:145. 10.1186/s13059-019-1762-8 31349852 PMC6660936

[130] Shang W Wang F Fan G Wang H. Key elements for designing and performing a CRISPR/Cas9-based genetic screen. J Genet Genomics. (2017) 44:439–49. 10.1016/j.jgg.2017.09.005 28967615

[131] Ford K Mcdonald D Mali P. Functional genomics via CRISPR-Cas. J Mol Biol. (2019) 431:48–65. 10.1016/j.jmb.2018.06.034 29959923 PMC6309720

[132] Robinson MD Mccarthy DJ Smyth GK. edgeR: a bioconductor package for differential expression analysis of digital gene expression data. Bioinformatics. (2010) 26:139–40. 10.1093/bioinformatics/btp616 19910308 PMC2796818

[133] Hardcastle TJ Kelly KA. baySeq: empirical bayesian methods for identifying differential expression in sequence count data. BMC Bioinformatics. (2010) 11:422. 10.1186/1471-2105-11-422 20698981 PMC2928208

[134] Love MI Huber W Anders S. Moderated estimation of fold change and dispersion for RNA-seq data with DESeq2. Genome Biol. (2014) 15:550. 10.1186/s13059-014-0550-8 25516281 PMC4302049

[135] Luo B Cheung HW Subramanian A Sharifnia T Okamoto M Yang X. Highly parallel identification of essential genes in cancer cells. Proc Natl Acad Sci USA. (2008) 105:20380–5. 10.1073/pnas.0810485105 19091943 PMC2629277

[136] Konig R Chiang CY Tu BP Yan SF Dejesus PD Romero A. A probability-based approach for the analysis of large-scale RNAi screens. Nat Methods. (2007) 4:847–9. 10.1038/nmeth1089 17828270

[137] Wang B Wang M Zhang W Xiao T Chen CH Wu A. Integrative analysis of pooled CRISPR genetic screens using MAGeCKFlute. Nat Protoc. (2019) 14:756–80. 10.1038/s41596-018-0113-7 30710114 PMC6862721

[138] Diaz AA Qin H Ramalho-Santos M Song JS. HiTSelect: a comprehensive tool for high-complexity-pooled screen analysis. Nucleic Acids Res. (2015) 43:e16. 10.1093/nar/gku1197 25428347 PMC4330337

[139] Yu J Silva J Califano A. ScreenBEAM: a novel meta-analysis algorithm for functional genomics screens via bayesian hierarchical modeling. Bioinformatics. (2016) 32:260–7. 10.1093/bioinformatics/btv556 26415723 PMC4907394

[140] Hart T Moffat J. BAGEL: a computational framework for identifying essential genes from pooled library screens. BMC Bioinformatics. (2016) 17:164. 10.1186/s12859-016-1015-8 27083490 PMC4833918

[141] Trumbach D Pfeiffer S Poppe M Scherb H Doll S Wurst W. ENCoRE: an efficient software for CRISPR screens identifies new players in extrinsic apoptosis. BMC Genomics. (2017) 18:905. 10.1186/s12864-017-4285-2 29178829 PMC5702081

[142] Jia G Wang X Xiao G. A permutation-based non-parametric analysis of CRISPR screen data. BMC Genomics. (2017) 18:545. 10.1186/s12864-017-3938-5 28724352 PMC5518132

[143] Allen F Behan F Khodak A Iorio F Yusa K Garnett M. JACKS: joint analysis of CRISPR/Cas9 knockout screens. Genome Res. (2019) 29:464–71. 10.1101/gr.238923.118 30674557 PMC6396427

[144] Tsherniak A Vazquez F Montgomery PG Weir BA Kryukov G Cowley GS Defining a cancer dependency map. Cell. (2017) 170:564–76.e516. 10.1016/j.cell.2017.06.010 PMC5667678 28753430

[145] Daley TP Lin Z Lin X Liu Y Wong WH Qi LS. CRISPhieRmix: a hierarchical mixture model for CRISPR pooled screens. Genome Biol. (2018) 19:159. 10.1186/s13059-018-1538-6 30296940 PMC6176515

[146] Jeong HH Kim SY Rousseaux MWC Zoghbi HY Liu Z. Beta-binomial modeling of CRISPR pooled screen data identifies target genes with greater sensitivity and fewer false negatives. Genome Res. (2019) 29:999–1008. 10.1101/gr.245571.118 31015259 PMC6581060

[147] Li W Xu H Xiao T Cong L Love MI Zhang F. MAGeCK enables robust identification of essential genes from genome-scale CRISPR/Cas9 knockout screens. Genome Biol. (2014) 15:554. 10.1186/s13059-014-0554-4 25476604 PMC4290824

[148] Yang L Zhu Y Yu H Cheng X Chen S Chu Y. scMAGeCK links genotypes with multiple phenotypes in single-cell CRISPR screens. Genome Biol. (2020) 21:19. 10.1186/s13059-020-1928-4 31980032 PMC6979386

[149] Li W Koster J Xu H Chen CH Xiao T Liu JS. Quality control, modeling, and visualization of CRISPR screens with MAGeCK-VISPR. Genome Biol. (2015) 16:281. 10.1186/s13059-015-0843-6 26673418 PMC4699372

[150] Chen CH Xiao T Xu H Jiang P Meyer CA Li W. Improved design and analysis of CRISPR knockout screens. Bioinformatics. (2018) 34:4095–101. 10.1093/bioinformatics/bty450 29868757 PMC6247926

[151] Chen W Zhang G Li J Zhang X Huang S Xiang S. CRISPRlnc: a manually curated database of validated sgRNAs for lncRNAs. Nucleic Acids Res. (2019) 47:D63–8. 10.1093/nar/gky904 30285246 PMC6324000

[152] Szlachta K Kuscu C Tufan T Adair SJ Shang S Michaels AD. CRISPR knockout screening identifies combinatorial drug targets in pancreatic cancer and models cellular drug response. Nat Commun. (2018) 9:4275. 10.1038/s41467-018-06676-2 30323222 PMC6189038

[153] Arroyo JD Jourdain AA Calvo SE Ballarano CA Doench JG Root DE. A genome-wide CRISPR death screen identifies genes essential for oxidative phosphorylation. Cell Metab. (2016) 24:875–85. 10.1016/j.cmet.2016.08.017 27667664 PMC5474757

[154] Ghezraoui H Piganeau M Renouf B Renaud JB Sallmyr A Ruis B. Chromosomal translocations in human cells are generated by canonical nonhomologous end-joining. Mol Cell. (2014) 55:829–42. 10.1016/j.molcel.2014.08.002 25201414 PMC4398060

[155] Ye L Wang C Hong L Sun N Chen D Chen S. Programmable DNA repair with CRISPRa/i enhanced homology-directed repair efficiency with a single Cas9. Cell Discov. (2018) 4:46. 10.1038/s41421-018-0049-7 30062046 PMC6056518

[156] Sakuma T Nakade S Sakane Y Suzuki KT Yamamoto T. MMEJ-assisted gene knock-in using TALENs and CRISPR-Cas9 with the PITCh systems. Nat Protoc. (2016) 11:118–33. 10.1038/nprot.2015.140 26678082

[157] Shen MW Arbab M Hsu JY Worstell D Culbertson SJ Krabbe O. Predictable and precise template-free CRISPR editing of pathogenic variants. Nature. (2018) 563:646–51. 10.1038/s41586-018-0686-x 30405244 PMC6517069

[158] Allen F Crepaldi L Alsinet C Strong AJ Kleshchevnikov V De Angeli P. Predicting the mutations generated by repair of Cas9-induced double-strand breaks. Nat Biotechnol. (2018) 37:64–72. 10.1038/nbt.4317 30480667 PMC6949135

[159] Chen W Mckenna A Schreiber J Haeussler M Yin Y Agarwal V. Massively parallel profiling and predictive modeling of the outcomes of CRISPR/Cas9-mediated double-strand break repair. Nucleic Acids Res. (2019) 47:7989–8003. 10.1093/nar/gkz487 31165867 PMC6735782

[160] Connelly JP Pruett-Miller SM. CRIS.py: a versatile and high-throughput analysis program for CRISPR-based genome editing. Sci Rep. (2019) 9:4194. 10.1038/s41598-019-40896-w 30862905 PMC6414496

[161] Wang X Tilford C Neuhaus I Mintier G Guo Q Feder JN. CRISPR-DAV: CRISPR NGS data analysis and visualization pipeline. Bioinformatics. (2017) 33:3811–2. 10.1093/bioinformatics/btx518 28961906

[162] Guell M Yang L Church GM. Genome editing assessment using CRISPR genome analyzer (CRISPR-GA). Bioinformatics. (2014) 30:2968–70. 10.1093/bioinformatics/btu427 24990609 PMC4184265

[163] Lindsay H Burger A Biyong B Felker A Hess C Zaugg J. CrispRVariants charts the mutation spectrum of genome engineering experiments. Nat Biotechnol. (2016) 34:701–2. 10.1038/nbt.3628 27404876

[164] Varshney GK Pei W Lafave MC Idol J Xu L Gallardo V. High-throughput gene targeting and phenotyping in zebrafish using CRISPR/Cas9. Genome Res. (2015) 25:1030–42. 10.1101/gr.186379.114 26048245 PMC4484386

[165] Clement K Rees H Canver MC Gehrke JM Farouni R Hsu JY. CRISPResso2 provides accurate and rapid genome editing sequence analysis. Nat Biotechnol. (2019) 37:224–6. 10.1038/s41587-019-0032-3 30809026 PMC6533916

[166] Boel A Steyaert W De Rocker N Menten B Callewaert B De Paepe A. BATCH-GE: batch analysis of next-generation sequencing data for genome editing assessment. Sci Rep. (2016) 6:30330. 10.1038/srep30330 27461955 PMC4962088

[167] Komor AC Kim YB Packer MS Zuris JA Liu DR. Programmable editing of a target base in genomic DNA without double-stranded DNA cleavage. Nature. (2016) 533:420–4. 10.1038/nature17946 27096365 PMC4873371

[168] Rees HA Liu DR Base editing: precision chemistry on the genome and transcriptome of living cells. Nat Rev Genet. (2018) 19:770–88. 10.1038/s41576-018-0059-1 30323312 PMC6535181

[169] Park J Lim K Kim JS Bae S. Cas-analyzer: an online tool for assessing genome editing results using NGS data. Bioinformatics. (2017) 33:286–8. 10.1093/bioinformatics/btw561 27559154 PMC5254075

[170] You Q Zhong Z Ren Q Hassan F Zhang Y Zhang T. CRISPRMatch: an automatic calculation and visualization tool for high-throughput CRISPR genome-editing data analysis. Int J Biol Sci. (2018) 14:858–62. 10.7150/ijbs.24581 29989077 PMC6036748

[171] Varshney GK Zhang S Pei W Adomako-Ankomah A Fohtung J Schaffer K. CRISPRz: a database of zebrafish validated sgRNAs. Nucleic Acids Res. (2016) 44:D822–6. 10.1093/nar/gkv998 26438539 PMC4702947

[172] Kaur K Tandon H Gupta AK Kumar M. CrisprGE: a central hub of CRISPR/Cas-based genome editing. Database. (2015) 2015:bav055. 10.1093/database/bav055 26120138 PMC4483309

[173] Rauscher B Heigwer F Breinig M Winter J Boutros M. GenomeCRISPR - a database for high-throughput CRISPR/Cas9 screens. Nucleic Acids Res. (2017) 45:D679–86. 10.1093/nar/gkw997 27789686 PMC5210668

[174] Lenoir WF Lim TL Hart T. PICKLES: the database of pooled *in-vitro* CRISPR knockout library essentiality screens. Nucleic Acids Res. (2018) 46:D776–80. 10.1093/nar/gkx993 29077937 PMC5753353

[175] Behan FM Iorio F Picco G Goncalves E Beaver CM Migliardi G. Prioritization of cancer therapeutic targets using CRISPR-Cas9 screens. Nature. (2019) 568:511–6. 10.1038/s41586-019-1103-9 30971826

[176] Oughtred R Stark C Breitkreutz BJ Rust J Boucher L Chang C. The BioGRID interaction database: 2019 update. Nucleic Acids Res. (2019) 47:D529–41. 10.1093/nar/gky1079 30476227 PMC6324058

[177] Dong C Hao GF Hua HL Liu S Labena AA Chai G. Anti-CRISPRdb: a comprehensive online resource for anti-CRISPR proteins. Nucleic Acids Res. (2018) 46:D393–8. 10.1093/nar/gkx835 29036676 PMC5753274

[178] Zhang F Zhao S Ren C Zhu Y Zhou H Lai Y. CRISPRminer is a knowledge base for exploring CRISPR-Cas systems in microbe and phage interactions. Commun Biol. (2018) 1:180. 10.1038/s42003-018-0184-6 30393777 PMC6208339

[179] Shin J Jiang F Liu JJ Bray NL Rauch BJ Baik SH. Disabling Cas9 by an anti-CRISPR DNA mimic. Sci Adv. (2017) 3:e1701620. 10.1126/sciadv.1701620 28706995 PMC5507636

[180] Anzalone AV Randolph PB Davis JR Sousa AA Koblan LW Levy JM Search-and-replace genome editing without double-strand breaks or donor DNA. Nature. (2019) 576:149–57. 10.1038/s41586-019-1711-4 31634902 PMC6907074

[181] Jayavaradhan R Pillis DM Goodman M Zhang F Zhang Y Andreassen PR. CRISPR-Cas9 fusion to dominant-negative 53BP1 enhances HDR and inhibits NHEJ specifically at Cas9 target sites. Nat Commun. (2019) 10:2866. 10.1038/s41467-019-10735-7 31253785 PMC6598984

